# Experimental bacterial adaptation to the zebrafish gut reveals a primary role for immigration

**DOI:** 10.1371/journal.pbio.2006893

**Published:** 2018-12-10

**Authors:** Catherine D. Robinson, Helena S. Klein, Kyleah D. Murphy, Raghuveer Parthasarathy, Karen Guillemin, Brendan J. M. Bohannan

**Affiliations:** 1 Institute of Molecular Biology, University of Oregon, Eugene, Oregon, United States of America; 2 Department of Physics and Materials Science Institute, University of Oregon, Eugene, Oregon, United States of America; 3 Humans and the Microbiome Program, Canadian Institute for Advanced Research, Toronto, Ontario, Canada; 4 Institute of Ecology and Evolution, University of Oregon, Eugene, Oregon, United States of America; Massachusetts Institute of Technology, United States of America

## Abstract

All animals live in intimate association with microorganisms that profoundly influence their health and development, yet the traits that allow microorganisms to establish and maintain host associations are not well understood. To date, most investigations aimed at identifying traits required for host association have focused on intrahost niches. Consequently, little is known about the relative contribution of extrahost factors such as environmental growth and survival and immigration into hosts from the external environment, as promoters of host association. To address this, we developed a tractable experimental evolution system that investigates both intra- and extrahost factors contributing to bacterial adaptation to the vertebrate gut. We passaged replicate lines of a zebrafish bacterial isolate, *Aeromonas veronii*, through populations of germ-free larval zebrafish (*Danio rerio*), each time using gut-associated *Aeromonas* populations to inoculate the aquatic environment of the next zebrafish population. We observed rapid increased adaptation to the host in all replicate lines. The initial adaptations present in early-evolved isolates did not increase intrahost fitness but rather enhanced both immigration from the environment and interhost transmission. Only in later-evolved isolates did we find evidence for intrahost-specific adaptations, as demonstrated by comparing their competitive fitness in the host genotype to which they evolved to that in a different genotype. Our results show how selection for bacterial transmission between hosts and their environment can shape bacterial-host association. This work illuminates the nature of selective forces present in host–microbe systems and reveals specific mechanisms of increased host association. Furthermore, our findings demonstrate that the entire host–microbe–environment system must be considered when identifying microbial traits that contribute to host adaptation.

## Author summary

Animals live in a world teeming with microbes; from this pool, unique subsets of microbes colonize animal tissues. We know very little about the selection pressures that contribute to a microbe’s ability to be host associated (i.e., to colonize or otherwise exist within a host). We used the zebrafish as a model animal host to investigate how a bacterium, *Aeromonas veronii*, increases its ability to be host associated. Our selection regime consisted of serially exposing larval zebrafish to *A*. *veronii*, each time selecting for gut-associated *A*. *veronii* populations. We found that the primary adaptive strategy of the bacterium was to increase immigration from the environment into the host. Selection to conditions specifically within the host only arose later in the experiment. The conditions within a host are often assumed to provide the primary selection for host association. Our results show that previously underappreciated aspects of host–microbe systems (e.g., immigration from the external environment) can play important roles in the establishment and maintenance of host–microbe relationships and that host–microbe interactions occur in a broader ecological context not generally captured in experimental models.

## Introduction

Animals are intimately associated with highly complex and dynamic communities of microbes that inhabit virtually every surface of their bodies. An explosion of research in recent decades has revealed that both the microbiota and the hosts they colonize have profound influences on the physiology and evolution of one another [1,2]. In vertebrates, it is especially evident that host–microbiota interactions within the gastrointestinal tract are fundamental to host health and development [3]. Wide-ranging surveys of the earth’s microbial communities have shown that host-associated microbial communities are distinct from non-host-associated communities, suggesting that there are unique properties of host–microbe systems that drive microbiome assembly [4]. Elucidating the microbial traits that allow host colonization and support host–microbe association could shed light on the ecology of host-associated microbes across the entire symbiotic spectrum, from mutualism (e.g., beneficial microbes) to parasitism (e.g., pathogens). Knowledge of these traits may also provide insights into the development of microbiota-driven diseases and ultimately into innovative ways to treat or prevent them.

Several different approaches have been used in past studies to identify traits that mediate microbial association with vertebrate hosts, including forward genetic screens (e.g., transposon mutagenesis) [5–8], functional genomics (e.g., transcriptomics) [9,10], and approaches from comparative genomics [11–13]. However, these approaches all have substantial limitations; for example, comparative genomics approaches have limited ability to distinguish between differences arising from adaptation to the host and those accumulated over evolutionary time in the non-host environment. Here, we use experimental evolution, which allows for the observation of evolution in real time under controlled laboratory conditions, to identify traits that mediate host association. This approach has the advantage that changes in traits can be directly attributed to the selection imposed by the experimental conditions, and there is no inherent constraint on the spectrum of mutations or traits that can be selected. Furthermore, this approach focuses on a phenotypic-level characterization of the system; adaptive traits may be acquired through many different genetic mechanisms, but their shared phenotypes reveal the selective pressures that drive their evolution.

Historically, experimental evolution has been a powerful strategy to study how microorganisms adapt to in vitro environments [14,15]. In recent years, this approach has been expanded to in vivo studies of host–microbe interactions, although it has been underutilized, relative to other approaches, for such questions [16]. Previous work has focused primarily on experimentally evolving microbes to the in vivo environment of a single host [17–21]. However, hosts and their associated microbes do not exist in isolation but rather within a broader ecological framework, and host association likely involves not just intrahost factors but also extrahost factors such as immigration into hosts from the external environment, host-to-host transmission, and extrahost growth and survival [22]. Indeed, previous work implicating the importance of dispersal and transmission in shaping host-associated microbial communities indicates that microbes adapt to more than just the within-host environment [23–29].

The zebrafish host–microbe model system is well suited to studying both intra- and extrahost factors simultaneously [30]. To perform precise, manipulative studies, there are established gnotobiotic protocols for generating germ-free (GF) zebrafish that can be colonized with defined microbial constituents [31]. Bacterial colonization of larval zebrafish occurs initially via the fish culture medium, which serves as a conduit supporting bacterial transmission throughout the system. Furthermore, tracking this transmission between the intra- and extrahost bacterial populations, as well as from host to host, is feasible. For example, previous work has demonstrated that gut populations can undergo dramatic expulsion events and that environmental populations contribute to repopulation of the gut [32].

**Fig 1:**
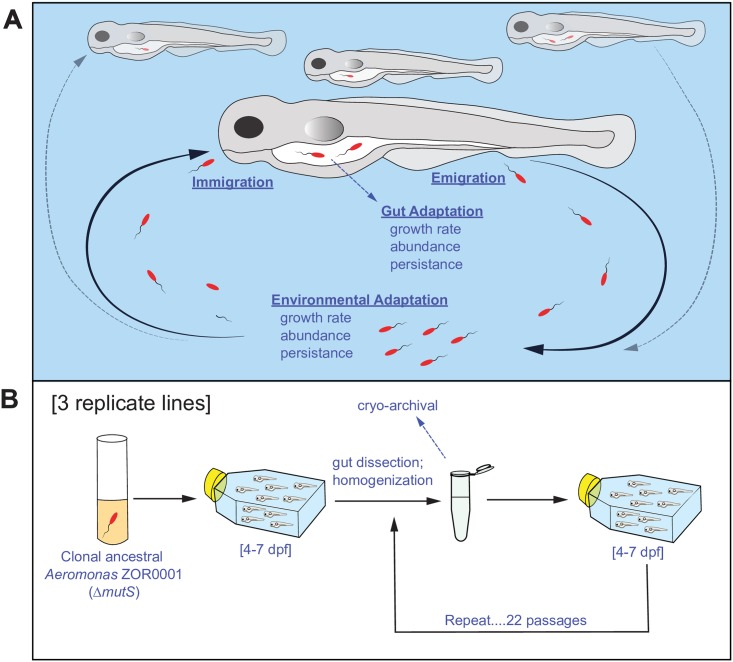
Experimental evolution of *Aeromonas* ZOR0001 in the larval zebrafish host. (A) Conceptual diagram of the colonization cycle and potential bacterial strategies for adaptation to the zebrafish gut. (B) Passaging scheme of the evolution experiment. dpf, days post fertilization.

Using this powerful model system, we conducted an evolution experiment by serially passaging the bacterium *Aeromonas* ZOR0001 through GF larval zebrafish hosts, each time specifically using gut-associated populations as inoculum for the subsequent passage. We found that host adaptation occurred quickly and reproducibly across replicate lines. Phenotypic characterization of the early adaptations (i.e., those found in early-evolved isolates) showed that they were not specific to the intrahost environment but rather they enhanced initial colonization, specifically immigration. Finally, we show that later adaptations confer host specialization by comparing the fitness of “early-” and “further-evolved” isolates in the host genotype in which they evolved to that of a different host genotype. Our results demonstrate that the intrahost environment does not always play the dominant role in selection of host-associated traits but rather the entire colonization cycle (immigration, intrahost growth and survival, emigration, and extrahost growth and survival; illustrated in Fig 1A) must be considered when identifying traits that confer host association.

## Results

### Characterization of the bacterial colonization cycle and experimental design

#### Selection of the model bacterium

The ideal bacterium for our study would be one that is biologically relevant (i.e., routinely found in the host, important to the host’s biology, etc.) and for which we have prior knowledge of its colonization dynamics and evidence of its adaptive potential. For these reasons, we selected *Aeromonas veronii* (strain ZOR0001), hereafter referred to as Aer01, which was previously isolated from the zebrafish gut [33]. This bacterium has numerous advantages for our study. There is a high-quality reference genome for this strain and established genetic tools [34]. *Aeromonas* is a prevalent genus within the intestinal community of the zebrafish, present throughout all developmental time points previously surveyed [33]. Its biological relevance is further evidenced by previous work showing that members of this genus can reverse GF host traits in mono-association [35–37]. Colonization dynamics, including generation time, population bottlenecks, and population size, influence the pace of adaptation of evolving populations. Previous work with this isolate determined its in vivo growth rate and average carrying capacity [32,38] and demonstrated that intestinal populations undergo dramatic population collapse and regrowth events, on average about once per day [32]. Finally, prior studies showing that this strain is readily outcompeted by other zebrafish gut isolates and that its average gut colonization level is below that of many other strains suggested that it had potential for further host adaptation [32,38].

The amount of genetic variation within a system is an additional parameter that strongly affects the rate of adaptation. Mutator strains carry mutations in the DNA replication or repair machinery, effectively increasing spontaneous mutation rates and, therefore, adaptive potential [39]. To increase the rate of adaptation in our system, we generated a mutator (*ΔmutS*) strain of Aer01. MutS is a component of the methyl-directed DNA mismatch repair system, and *mutS* mutants commonly arise in experimental evolution studies [39–41] and naturally evolving systems [42,43]. Allelic exchange was used to make an in-frame deletion in the *mutS* gene of a fluorescently tagged Aer01 strain. A fluctuation assay confirmed that the *mutS* mutant had an approximately 1,000-fold-increased mutation rate compared to the wild-type (WT) strain (S1 Table).

The trade-off for increasing adaptive potential by using a mutator strain is that background accumulation of mutations confounds identification of adaptive alleles. Since the primary goal of this study was to identify traits that confer host association, rather than the underlying genes, we chose to utilize the mutator phenotype as a way of accelerating evolution and facilitating adaptation in our model system.

#### Characterization of the colonization cycle

We expanded on previous work investigating Aer01 colonization dynamics by further characterizing key features of the Aer01 colonization cycle in our model system. Larval zebrafish hosts are inoculated by adding Aer01 to the water column, which consists of sterile embryo medium (EM), and thus, colonization begins with Aer01 immigrating into the host. We first determined the robustness of Aer01 colonization as a function of inoculum concentration, which could fluctuate during the evolution experiment. Gut colonization levels were not affected by inoculum size (over >4 log range), consistently reaching about 10^4^ colony-forming units per gut (CFU/gut; S1A Fig). However, we found that independent of starting concentration, Aer01 is detected in the EM at about 10^5^ CFU/ml by 20 hours post inoculation (S1B Fig). These data show that there is a defined carrying capacity both for populations in the host gut and also in the extrahost environment.

Additional colonization parameters important for adaptation in this system are (1) the size of the initial colonizing population and (2) the frequency of mixing between the extra- and intrahost environments over time. These are important because they influence the pool of genetic variants that will be sampled within this model system, which impacts the rate of adaptation. To estimate the size of the initial (first 24 hours of colonization) bottleneck, we investigated the colonization success of a tagged strain of Aer01 in the gut when introduced at various low frequencies in the inoculum. We found that when a 1:100 mixture of dTomato*-*tagged Aer01 and WT Aer01 was added to the external medium, at 24 hours post inoculation the tagged strain was detected in 80% of fish (*n* = 54), whereas at a ratio of 1:300 it was only detected in 38% (*n* = 53) (S2 Fig). We used a binomial sampling model (see Materials and methods) to estimate a 24-hour bottleneck size of 194 ± 140 Aer01 cells, demonstrating that the founder population in a fish gut is composed of much more than a few cells but is still multiple orders of magnitude below the average gut colonization level.

Once the gut population reaches carrying capacity, there may be additional influx as bacteria are expelled from the intestinal tract and new cells migrate in from the environment. The ability of Aer01 to invade already-established populations has previously been reported [32]. We verified this finding and confirmed that members of a differentially tagged Aer01 population, when inoculated into a flask of precolonized Aer01 fish, are present in the gut after 24 hours, at an average ratio of about 2:1 (founder:invader) (S3 Fig). These data show that the larval host is continually sampling from environmental populations over time.

Larval zebrafish undergo rapid development over the 3-day time frame (4–7 days post fertilization [dpf]) during which we conducted our colonization experiments. We wanted to determine, at a broad level, if Aer01 colonization is affected by the stage of host development. To test this, we colonized fish at 4, 5, or 6 dpf and enumerated bacterial load after 24 hours by gut dissection and plating. The average CFU/gut was not significantly different across the three time points, demonstrating that Aer01 colonization level is consistent across these host developmental windows (S4 Fig).

These results provide a detailed picture of the Aer01 colonization cycle in our model system (Fig 1A). This cycle provides multiple opportunities for Aer01 to increase host association (i.e., the chance of being found in the gut). Gut-specific adaptations include increased growth rate, increased abundance (potentially via increased carrying capacity), and increased persistence via tolerance to stresses within the host environment, or resistance to expulsion by peristaltic activity of the gut. Adaptations related to environmental growth, abundance, or persistence could also increase host association because they would increase the probability of being sampled by the host throughout colonization. Finally, changes in the capacity for migration, either increased immigration or decreased emigration, could also improve host association.

#### Evolution passaging scheme

To identify traits that promote host association in our system, we serially passaged three replicate clonal mutator Aer01 “ancestral” populations through groups of 10–15 WT larval zebrafish (Fig 1B). For each passage, the Aer01 populations were inoculated into flasks of GF 4 dpf larval fish containing EM (the external medium in our system). After 3 days (i.e., 7 dpf), the fish were euthanized and their guts harvested by dissection to specifically select for gut-associated Aer01 populations. The guts were combined and homogenized to release the bacteria; then, an aliquot (approximately half of the total sample) was used as inoculum for the next group of 4 dpf GF larval fish, and the remaining gut sample was cryopreserved. This process was repeated for 22 passages.

### Evolutionary adaptation of Aer01 to the larval zebrafish gut occurred quickly and reproducibly across replicate lines

**Fig 2:**
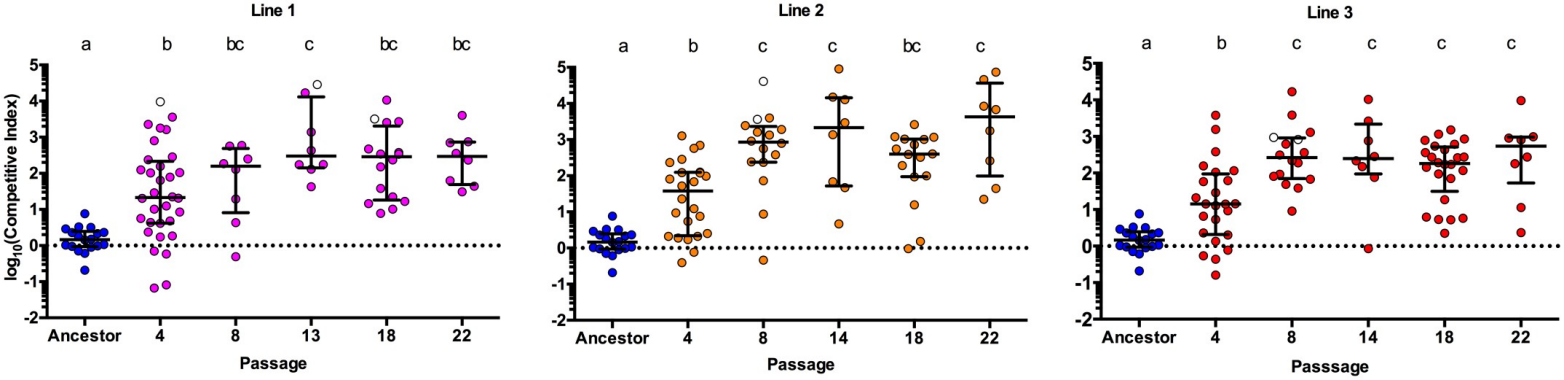
Adaptation of Aer01 occurred quickly and reproducibly across replicate lines. CIs of the test strain (evolved isolates or the ancestor) when competed against a differentially tagged ancestral reference strain in the larval fish. CI = (test/reference)_end_/(test/reference)_start_. Each circle represents a CI from an individual fish. Open circles represent data points at which the reference strain was undetected on plates; therefore, CIs were calculated by setting the abundance of the reference strain to the limit of detection (5 CFU/gut), thus providing a lower bound for the CI for these points. Ancestral data are the same in all plots. Median and interquartile ranges are plotted for all data. Conditions that share a letter are not statistically different (ANOVA, log-transformed data). *n* ≥ 8 fish/condition. Dotted line indicates CI value in the absence of any competitive advantage. Underlying data are provided in S1 Data. CFU/gut, colony-forming unit per gut; CI, competitive index.

Cryo-archived evolved population samples from passages 4, 8, 13/14, 18, and 22 were streaked for isolation on rich media, and freezer stocks of colony-purified isolates were made. These isolates were assayed for adaptation by competing them against a non-mutator, differentially tagged (green fluorescent protein [GFP]) Aer01 reference strain. For the competitions, ancestral or evolved isolates were mixed with the reference strain and inoculated into the EM of individual flasks of GF fish. Competitions were conducted under the same conditions as the evolution passages, for which strains were competed for 3 days (from 4 to 7 dpf), and all aspects of the colonization cycle are included. At 7 dpf, the fish were dissected and the guts plated to enumerate in vivo abundances of each strain via fluorescence microscopy. Competitive indices (CIs) were calculated by dividing the strain ratio (competitor:reference) at the end of the competition by the starting ratio (Fig 2). Adaptation occurred very quickly (by passage 4) in all three replicate lines, with greater than 10-fold average CIs for the passage 4 isolate from all three lines. This increase in competitive fitness is maintained or increased (approximately 100- to 1,000-fold averages) across isolates from later passages. The increased competitive fitness of the evolved isolates is not due to a general growth advantage, as there are no competitive advantages in vitro, in rich media (S5 Fig).

### The competitive advantage of early-evolved isolates is dependent on the mode of inoculation

**Fig 3:**
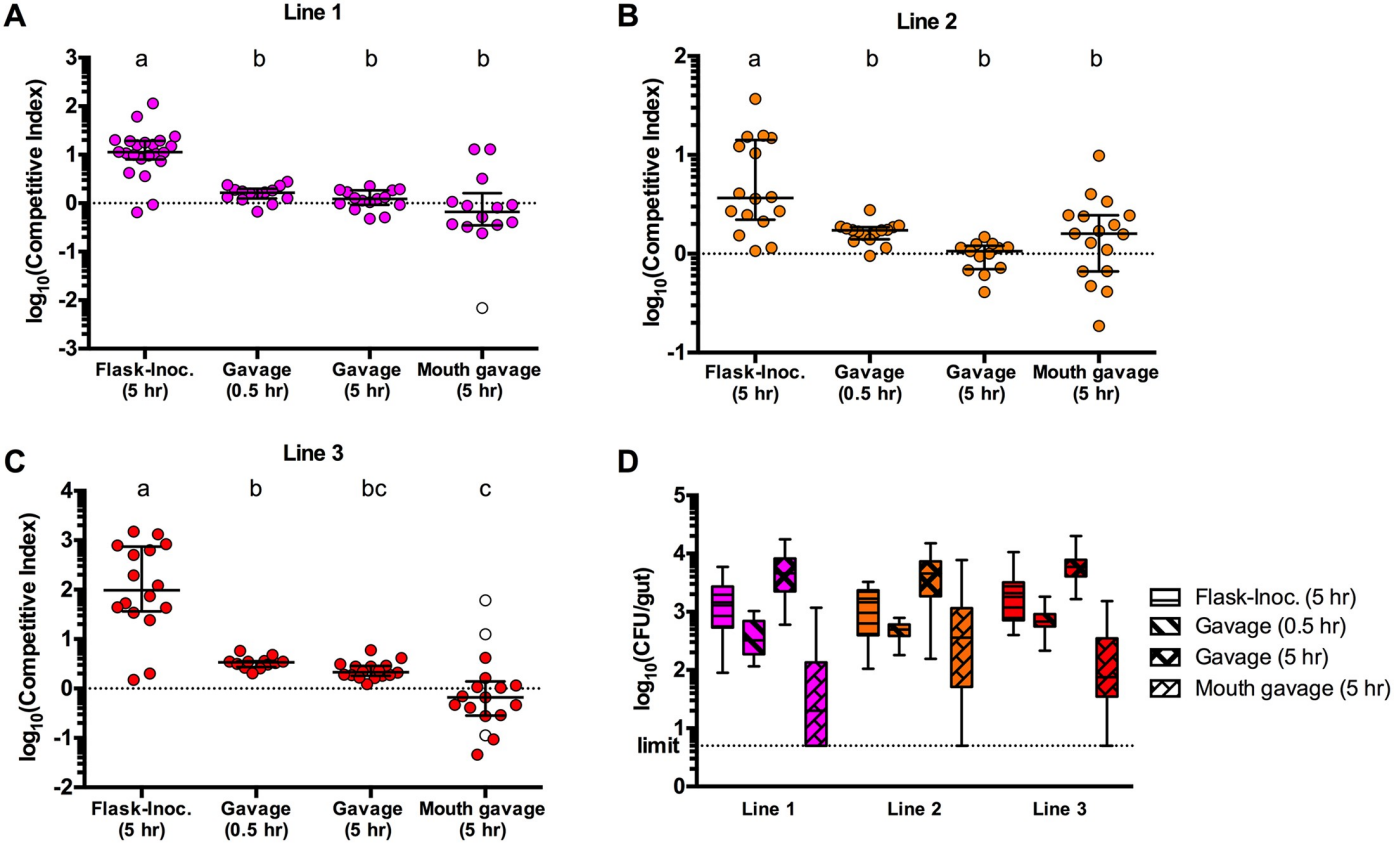
The competitive advantage of early-evolved isolates is dependent on mode of inoculation. (A–C) Competitive indices of passage 4 evolved isolates (A- line 1; B- line 2; C- line 3) when inoculated either via the flask EM or via gavage into the gut or mouth. Competitive indices were calculated based on plating data from guts dissected either 0.5 or 5 hours post inoculation, as indicated. Median and interquartile ranges are plotted for all data. (D) CFU/gut data from matched samples in panels A–C. For A–C, conditions that share a letter are not statistically different (ANOVA, log-transformed data). Data combined from two independent experiments; *n* ≥ 12/condition. Underlying data for A-D are provided in S1 Data. CFU/gut, colony-forming unit per gut; EM, embryo medium; Flask-Inoc., flask-inoculated.

To determine how the initial adaptations observed in the passage 4 isolates improved fitness, we next investigated the stage of the colonization cycle impacted. To do this, we used an inoculation approach that would isolate where competition takes place, thereby distinguishing fitness specific to the within-host environment from that of extrahost factors. Microgavaging is a technique whereby a blunt needle is used to deliver substances—in this case, bacterial cultures—directly into the gut via the oral route [44]. Competition mixtures of ancestral or passage 4 evolved isolates and reference strain (as above) were inoculated directly into the guts of GF larval fish. Concurrently, the same inocula were introduced into the flasks of GF fish as in the previous competition experiments. For all three lines, the competitive advantage of flask-inoculated evolved isolates was significantly diminished when the strains were introduced directly into the gut (Fig 3A–3C). This loss of competitive advantage was apparent at both 0.5 hours and 5 hours post gavage (Fig 3A–3C). The matched CFU/gut data for the same fish show that Aer01 was gavaged at sufficiently low densities (approximately 5 × 10^2^ CFU/gut) to allow for >10-fold increase in abundance during the 5-hour colonization (Fig 3D). If the CIs had increased between 0.5 and 5 hours post gavage, this would have indicated either a higher growth rate of the evolved strains or a better capacity to persist or survive within the gut environment. Since there were no differences in CIs between these time points, neither of these traits can be ascribed to the evolved lines. Instead, the competitive advantage of the evolved isolates is apparent when they are inoculated into the flasks (Fig 3A–3C), with median CIs of 11, 4, and 98 for evolved isolates from lines 1, 2, and 3, respectively. This suggests that the competitive advantage of these isolates is specific to initial colonization events, prior to reaching the gut environment. We wondered whether the competitive advantage was due to the ability to transmit from the mouth into the gut. To investigate this, we gavaged competition mixtures into the mouths of the fish rather than into the gut and then again measured the gut CIs after 5 hours. As with the gut gavages, the evolved strains when introduced into the mouth showed little to no significant advantage over the ancestral strain (Fig 3A–3C). In addition, the variation in these data is high, implying there is a variable—and at times tight—bottleneck imposed between the mouth and the gut such that in some fish one tag or the other dominates the *Aeromonas* gut population. Combined, these data suggest that the competitive advantage of the early-evolved isolates is driven by initial colonization of the host from the environment.

### Evolved isolates have higher rates of immigration into the host gut

**Fig 4:**
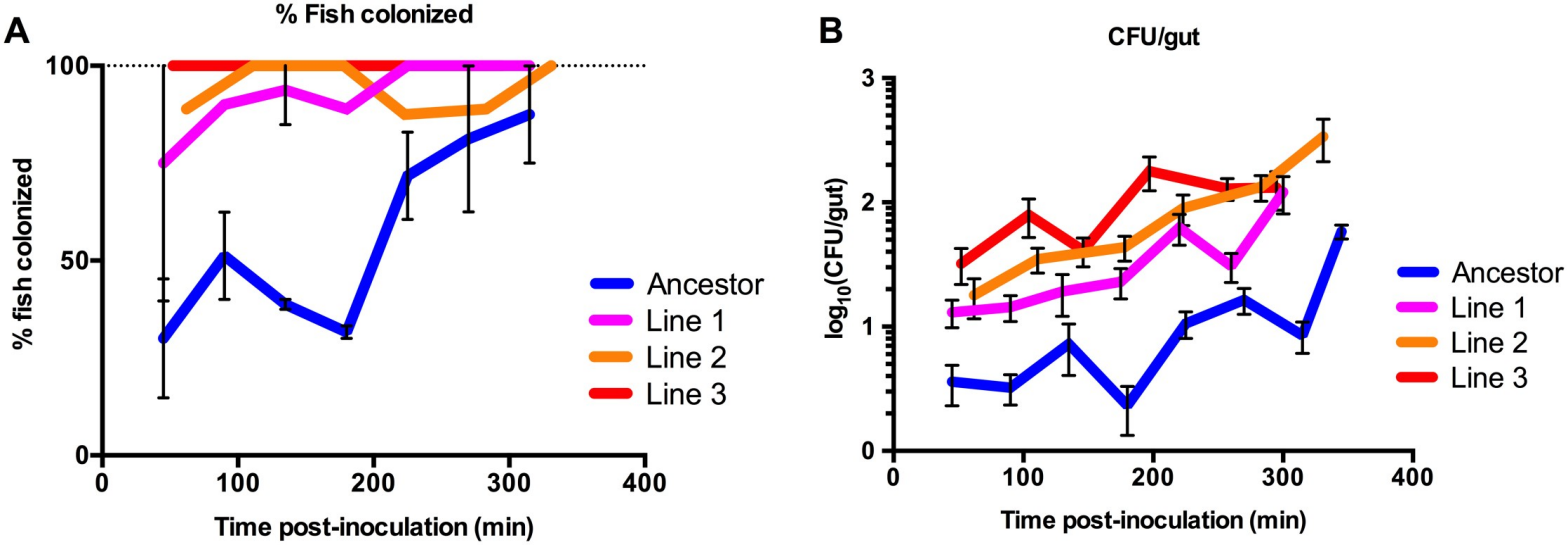
Evolved isolates demonstrate increased immigration into zebrafish hosts. (A) Groups of mono-associated fish were dissected and plated approximately every 45 minutes, and the fraction of colonized hosts was determined (*n* = 8–10 fish per time point). A higher proportion of fish were colonized at earlier time points for the evolved isolates compared to the ancestor. Means (± SEM) are plotted for ancestor and line 1. (B) CFU/gut data (mean ± SEM) from the same samples presented in A show higher gut abundance for the evolved isolates at all time points. Data combined from three (ancestor), two (evolved, line 1), or one (evolved, lines 2 and 3) independent experiments. Underlying data for A–B are provided in S1 Data. CFU/gut, colony-forming unit per gut.

To further quantify differences between passage 4 evolved isolates and the ancestral strain, we conducted migration rate experiments. Here, fish were mono-associated (inoculated into the flask) with either the ancestor or a passage 4 evolved isolate. Every 45 minutes, groups of 8–10 fish were dissected and the guts plated to determine (1) the fraction of fish colonized at each time point (Fig 4A) and (2) the *Aeromonas* abundance (Fig 4B). All three evolved isolates were present in a higher proportion of fish at each time point than the ancestor, with close to 100% colonization by the evolved isolates even at the earliest time points, whereas the ancestor was not present in 100% of the fish even at the final sampling (about 5 hours post inoculation). Abundance of the evolved isolates in the guts was also higher than the ancestral strain, with, on average, approximately 10-fold higher CFU/gut at the first time point compared to the ancestor. These data do not reflect a difference in growth or survival in the flask EM, since abundance remained relatively constant for all strains throughout the experiment (S6 Fig).

In addition to assessing bacterial colonization of zebrafish directly from the flask medium, we wanted to test if the increased migration phenotype of the evolved isolates could affect fish-to-fish colonization. To do this, we mono-associated fish with either the ancestor or the passage 4 isolate from line 3, washed and added two “donor” fish to a flask of GF “recipient” fish, and dissected and plated the recipient fish 12 hours later. Indeed, we found that the abundance of the evolved isolate was significantly higher than the ancestor in their respective recipient fish (S7 Fig).

We next developed a mathematical stochastic colonization and growth model (see Materials and methods for details) with which we could use the measured distribution of bacterial abundances across fish from the migration rate experiments to estimate the migration and growth rates of each strain. We consider a model in which migration into a fish by an individual bacterium is a stochastic event, with some probability per unit time. The statistics of the colonizers at any time are therefore governed by a Poisson distribution characterized by a mean time to entry, τ. Following entry into the host, the descendants of a colonizer obey logistic growth (initially roughly exponential, constrained by a finite carrying capacity, *K*), characterized by some growth rate *r*. We fit the experimental data (i.e., each set of CFU measurements at a particular measurement time *t*) to the above model of stochastic colonization and growth, determining the best-fit parameters τ and *r*. The datasets from each measurement time give independent estimates of *τ* and *r*; the average and standard error give the final estimates of mean entry time, growth rate, and their uncertainty for each bacterial strain (Table 1). We found that the characteristic time required to enter a larval zebrafish, *τ*, is approximately 2-fold smaller for the evolved isolates compared to the ancestor. Furthermore, the model output did not show significant differences in in vivo growth rates among these strains.

**Table 1:** Estimates for colonization time (*τ*) and in vivo growth rate (*r*) for ancestral and early-evolved (passage 4) isolates.

Strain	*τ* (SE), minutes	*r* (SE), 1/minute	*N*
Ancestor	130.0 (27.9)	0.0240 (0.0063)	9
Evolved, line 1	61.1 (8.6)	0.0260 (0.0059)	9
Evolved, line 2	60.0 (14.4)	0.0280 (0.0060)	6
Evolved, line 3	44.0 (7.5)	0.0268 (0.0053)	5

*N* = number of time points combined for determination of mean and SE.

Abbreviation: SE, standard error.

### Evolved isolates demonstrate increased motility in swimming assays

**Fig 5:**
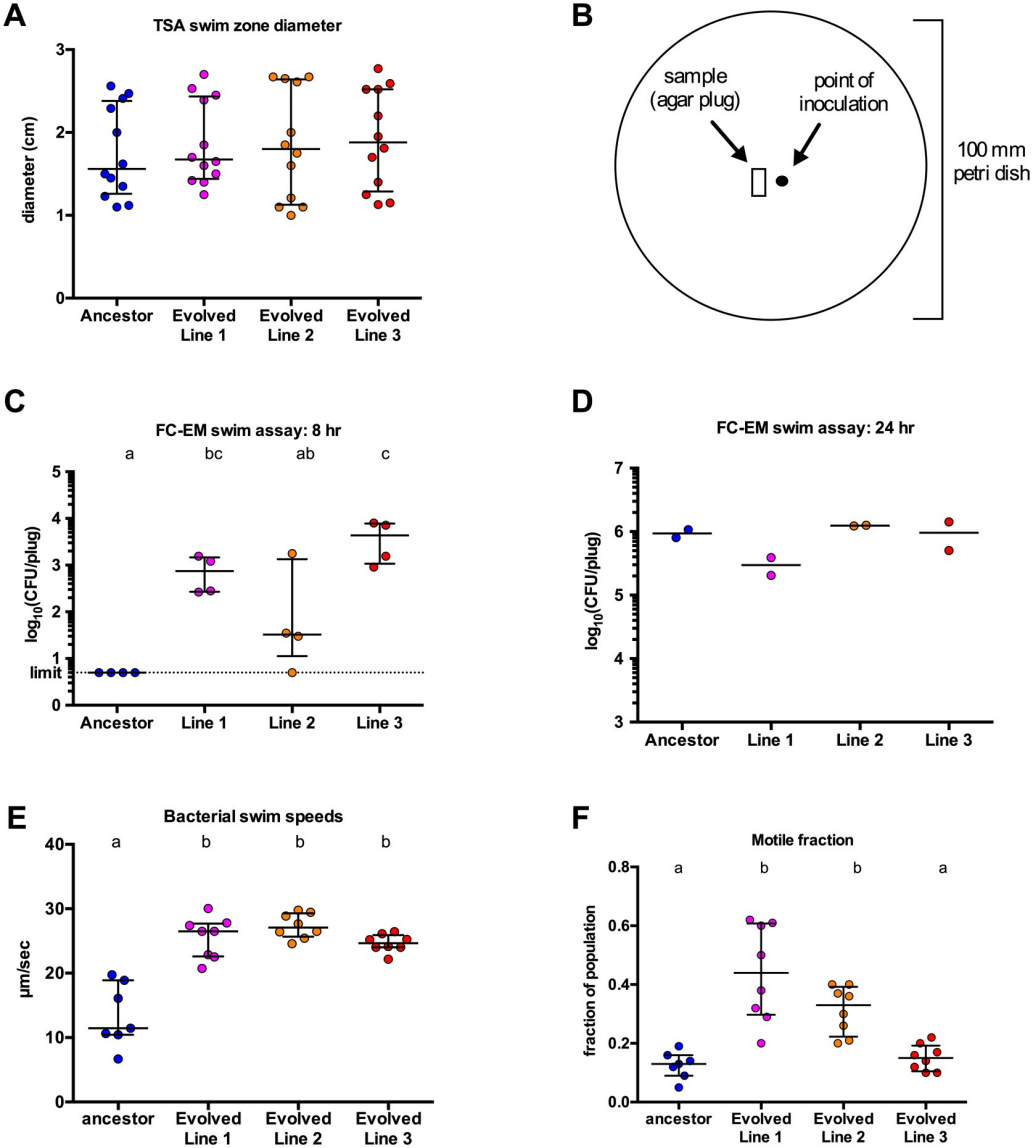
Evolved isolates demonstrate increased motility compared to the ancestor. (A) Diameter of swim zones at 8 hours post inoculation for swim plate assay in rich media (TSA). (B) Template for FC-EM swim plate inoculation and sampling; the black dot is the point of inoculation; the small rectangle represents the site of sampling via gel extractor tool. (C and D) Abundance of strains in samples from FC-EM swim plate assays; motility was assessed by sampling an agar plug and enumerating migrated cells via plating at 8 hours (C) or 24 hours (D). (E and F) Bacterial swim speeds (E) and motile fraction of the population (F) were measured directly via live imaging; each data point is representative of an individual movie in which an average of 6,000 (minimum of 2,300) bacterial cells were tracked. Median and interquartile ranges are plotted for all data. Conditions that share a letter are not statistically different (ANOVA, log-transformed data for C). Data combined from two independent experiments for all assays. Underlying data for A–F are provided in S1 Data. CFU/gut, colony-forming unit per gut; FC-EM, fish-conditioned embryo medium; TSA, tryptic soy agar.

In an effort to identify the physiological mechanism underlying increased migration into the host, we investigated the motility behavior of the ancestral and evolved isolates. The role of motility and chemotaxis in host associations is well recognized [5,45–48], especially for pathogenic bacteria [49,50]. Furthermore, the importance of motility and chemotaxis in the zebrafish–microbe symbiosis has been previously reported [5,46]. How these traits increase host colonization has not been characterized extensively, but they could play roles in increasing the rate of microbe–host contact. We conducted low-agar swim plate assays using rich media (tryptic soy broth [TSB]) or media made from fish-conditioned EM (FC-EM), which more closely replicates the conditions experienced by Aer01 when colonizing fish. Motility in the rich media plates was determined via standard protocol, by measuring the diameter of the motility zone. In rich media, population progression away from the site of inoculation is facilitated by growth, motility, and chemotaxis. We observed no significant differences in diameters of the motility zones between the ancestor and evolved isolates, indicating no motility advantage in rich media (Fig 5A). FC-EM was obtained by filter-sterilizing EM from flasks of hatched 5–6 dpf GF zebrafish larvae. It is essentially a nutrient-poor, dilute salt solution containing only limited fish-derived nutrients (mucus and other secreted or sloughed fish compounds), and therefore, the contribution of growth to progression through this media should be minimal. Cell density in FC-EM swim plates is too low to visually measure a motility zone. Hence, assessment of Aer01’s ability to swim in the FC-EM plates was accomplished by sampling an agar plug at a standardized distance away from the site of inoculation (Fig 5B). The plates were inoculated from rich media liquid cultures; then, a plug was sampled 8 hours and 24 hours post inoculation, homogenized, and plated to enumerate Aer01 cells. In FC-EM swim plates, a significantly higher number of cells were present in the agar plug at the 8 hour postinoculation sampling for each of the evolved isolate populations, compared to the ancestor (Fig 5C). Replicate FC-EM swim plates sampled at 24 hours show that the ancestor did eventually migrate to the standardized distance given sufficient time (Fig 5D).

Differences in progression of populations through the media in swim plate assays can be attributed to either chemotaxis (i.e., the ability to sense and respond to gradients of chemical attractants and/or repellants) or capacity for motility (e.g., presence of functional flagellar machinery, differences in cellular swim speeds, etc.). To distinguish between these and more precisely characterize the advantage of the evolved isolates, we used microscopy to directly track and measure cellular swimming characteristics of Aer01 ancestral and evolved populations. Ancestral and evolved isolates were incubated in FC-EM for several hours to acclimate and then were imaged. In these experiments, we were specifically testing for differences in general motility, as there are no chemical gradients and therefore no influence of chemotactic behavior. For each strain, 8 movies were recorded (from two independent replicate cultures), which were analyzed with custom tracking software to measure the velocity of thousands of cells each. The swim speed data are plotted in Fig 5E. Each dot represents the mean swim speed across the entire population of cells tracked in an individual movie. The ancestor had a median swim speed of 11.5 μm/second, whereas the evolved isolates from lines 1, 2, and 3 were about 2-fold faster, on average, with median swim speeds of 26.5, 27, and 24.7 μm/second, respectively (Fig 5E). When we determined the motile fraction of the population from each movie (i.e. the proportion of the cells tracked with >5 μm/second swim speeds), two of the three evolved isolates also had significantly higher motile fractions compared to the ancestor, with the ancestor populations displaying a median motile fraction of 0.13, whereas the medians for evolved isolates from lines 1 and 2 were 0.44 and 0.33, respectively (Fig 5F). Taken together, these data demonstrate that the evolved isolates have a hypermotile phenotype in this nutrient-poor medium, the same condition that Aer01 experiences in a fish flask.

### Further-evolved isolates have host genotype–dependent competitive fitness outcomes

The experiments described above were focused specifically on investigating the adaptation mechanisms of the earlier-evolved (passage 4) isolates. Based on the progressive increase in CIs seen in further-evolved isolates (Fig 2), we hypothesized that those strains carried adaptations in addition to the increased immigration phenotype seen in passage 4 isolates. We first verified that further-evolved isolates (passage 18) have the increased-immigration phenotype (S8 Fig; S2 Table). The two passage 18 isolates tested from two of the independent lines both had increased migration rates, of a similar magnitude as the passage 4 isolates, compared to the ancestor. This is consistent with the model that they are of the same lineage as the passage 4 isolates, carrying additional adaptive mutations, rather than being independent lineages with more beneficial gain-of-fitness mutations.

We then hypothesized that the further-evolved isolates have adaptations specific to the intrahost environment. Identification of a host genotype in which evolved isolates compete differently compared to the WT host would support our hypothesis that the evolved isolates are adapted to the specific conditions within the WT host.

**Fig 6:**
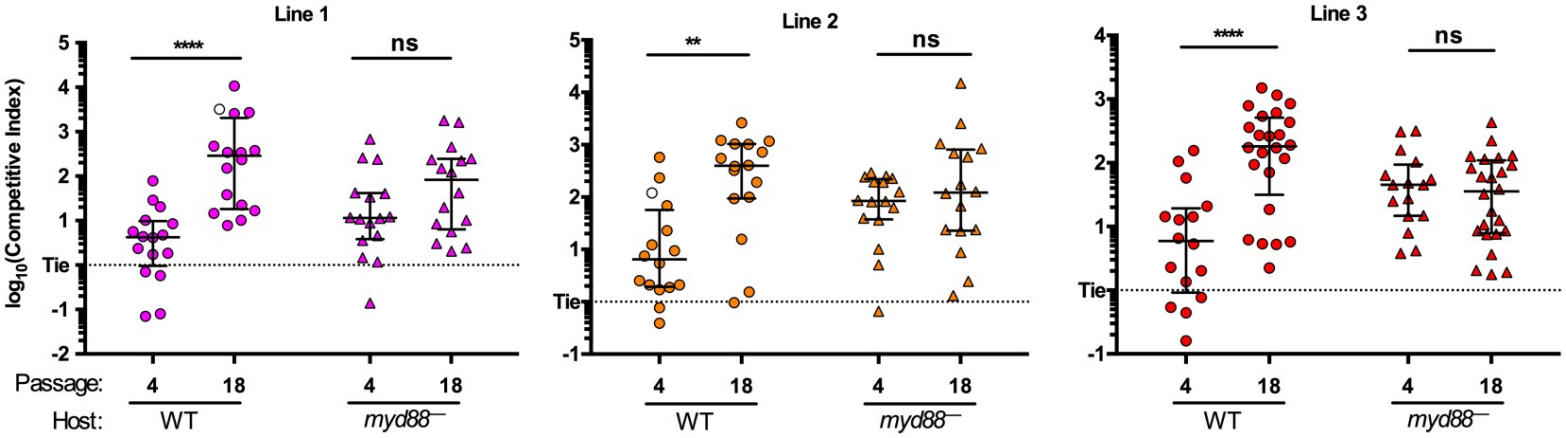
Further-evolved isolates have host genotype–dependent competitive fitness outcomes. CIs of evolved isolates and the ancestor, when competed against a differentially tagged ancestral strain in either WT (circles) or *myd88*^*—*^(triangles) larval zebrafish. Each data point represents a CI from an individual fish. Open symbols represent data points at which the reference strain was undetected on plates; therefore, CIs were calculated by setting the abundance of the reference strain to the limit of detection (5 CFU/gut), thus providing a lower bound for the CI for these points. ****p < .0001, **p < .01, *p < .05, ANOVA, log-transformed data, Tukey post hoc analysis. Data combined from two independent experiments for all assays (*n* ≥ 16/condition). Underlying data are provided in S1 Data. CFU/gut, colony-forming unit per gut; CI, competitive index; ns, not significant; WT, wild type.

To test this, we compared CIs when competed in WT hosts (the same host genotype used for the evolution experiment) to a different host genotype, an *myd88*^*—*^mutant. MyD88 is a protein adaptor for innate immune receptors, including the Toll-like receptors; hence, mutants are immunodeficient [23,51]. Use of this alternate host genotype is based on the rationale that host immune functions in the vertebrate gut potentially alter, either directly or indirectly, the gut environment [52,53]. Indeed, we have shown previously that *myd88* mutant larval zebrafish intestines differ from WT intestines in their paucity of neutrophils, mucus-secreting goblet cells, and proliferating epithelial cells [36,51,54]. We first verified that there were no overt differences in *Aeromonas* colonization of the *myd88*^*—*^fish by showing that ancestral Aer01 competed equally in both WT and *myd88*^*—*^hosts (S9 Fig). We then measured the CIs of early- and further-evolved isolates in both host genotypes. As in WT hosts, early-evolved isolates had increased CIs in *myd88*^*—*^hosts. The increased migration rate of early-evolved isolates was recapitulated in the *myd88*^*—*^host, confirming that the mechanism of this competitive advantage is the same as in WT fish (S10 Fig). For all three lines, the CIs of the passage 18 isolates in WT hosts are significantly higher than the passage 4 isolates in WT hosts (Fig 6). If this advantage was not specific to the host in vivo environment, it would be expected that the CIs of the passage 18 isolates in *myd88*^*—*^hosts would also be higher compared to the level observed in the passage 4 isolates in *myd88*^*—*^hosts. However, there were no significant differences in CIs in *myd88*^*—*^hosts between the passage 4 and passage 18 isolates. Combined, these data support that further-evolved isolates have additional adaptations specific to the intrahost environment, because they confer increased fitness in the WT host but do not provide fitness advantages in a different host genotype.

## Discussion

We have developed a new model system in which the roles of both intra- and extrahost environments can be studied simultaneously to investigate the microbial traits that contribute to host association. Using this system, we observed that Aer01 quickly and reproducibly increased in zebrafish host association and that the adaptive strategy of the early-evolved isolates was an increased ability to migrate into the host. This was first supported by data showing that competitive fitness is dependent on the mode of inoculation, such that the evolved isolates only out-competed the ancestral strain when inoculated through the flask water, as opposed to inoculating fish directly by gavage (Fig 3). By investigating the initial colonization dynamics of these isolates at a finer resolution, through sampling mono-associated fish at short time increments, we were then able to observe, quantify, and model this increased immigration (Fig 4; Table 1). This trait contributes to the within-host fitness of the evolved strains by enabling them to constitute a larger portion of the founding population within a host and, consequently, a larger portion of the mature population. Although the predominant impact of early adaptations is on immigration, we cannot rule out that there could be pleiotropic effects of these adaptions on other aspects of host colonization. For example, they could affect gut transit time or expulsion rates. Further investigation exploring the secondary impacts of early adaptions on colonization dynamics could provide additional biological insight into the mechanisms of host colonization.

### Immigration impacts multiple facets of colonization dynamics

In addition to playing a role in the initial establishment of the gut community, enhanced immigration could amplify the competitive fitness of the evolved isolates in intrahost populations over time. It has been shown previously that Aer01 goes through major stochastic population collapse events approximately every 24 hours, with decreases in abundance of more than 90% in as little as 30 minutes [32]. These collapse events result in Aer01 expulsion from the fish vent via gut motility and peristaltic activity. Recovery of the gut population from these events can occur from the residual population within the gut, but external Aer01 can also contribute to repopulation [32]. Indeed, we previously established that there is mixing of extrahost with intrahost populations over time (S3 Fig). Therefore, it is likely that our evolved, hypermigratory Aer01 strains have the potential to out-compete ancestral Aer01 for available open niche space during the repopulation phase that follows each collapse event. This amplification could explain how increased migration evolved so quickly within this system, because it would effectively ratchet up the strength of selection for the immigration phenotype. Moreover, these neutral processes (stochastic collapse events) combined with the deterministic processes (repopulation/regrowth dynamics) may account for the high variability we observe within our competition data. This combination of collapse and regrowth events is not unique to Aer01; these types of dynamics have been reported for other host-associated microbiome constituents—for example, from the human skin [55] and intestinal tract [56]. Whether these cyclic growth dynamics impart a selective pressure for transmission traits and contribute to the evolution of these communities is an open question.

Another way in which the immigration phenotype could impact colonization dynamics within this system is through host-to-host transmission. Our data showing that an evolved isolate had higher abundance than the ancestor in GF recipient fish when introduced from mono-associated donor fish support this hypothesis (S7 Fig). Recent research has demonstrated a role of transmission and dispersal in shaping the gut communities of many animals [23,26,57–60]. For example, Burns and colleagues showed that interhost dispersal is a key driver of gut community assembly in zebrafish hosts and can even override the selective forces imposed by the host immune system [23]. This body of literature suggests that selection of traits that improve transmission or dispersal among hosts could be a dominant force in the evolution of host-associated microbes. The work presented here provides support for this hypothesis and has important implications for how we think about the ways in which bacterial symbionts initiate and hone relationships with their hosts. In the future, it will be important to add back complexity to this distilled model system—for example, by increasing microbiota diversity—and determine if the relative importance of immigration is evident. Such studies could reveal trade-offs between colonization of the host and microbe–microbe competitive fitness.

Enhanced transmission is predicted to alter a number of important aspects of host–microbe ecology. For example, it has been proposed that trade-offs exist between transmission and virulence [61] and that mode of transmission influences virulence evolution [62]. However, we do not have any evidence for Aer01 virulence in zebrafish. Previous work has shown that Aer01, in mono-association, does not elicit a host immune response above what is measured in conventional fish and can even attenuate the host response to hyper-immunostimulatory species [38]. Of note, we did not observe any indications of pathogenicity in evolved, hypertransmissible Aer01.

In order to more thoroughly characterize the phenotype of our evolved isolates, we developed a mathematical model to estimate and compare the migration and in vivo growth rates of our ancestral and evolved isolates. Our mathematical model links observed population statistics to essential processes of bacterial colonization, treated stochastically, followed by logistic growth, treated deterministically. We note, however, that additional processes are present and could be elaborated in future models if warranted by relevant data. Bacterial growth, death, and expulsion from the gut [32] are inherently stochastic, and future stochastic models could quantitatively link these processes to measurable population statistics, either through brute force computation or potentially by making use of recently developed analytic tools [63]. Additional processes neglected in this work include the transition time between lag phase and exponential growth upon entering the gut environment (itself stochastic) and interindividual variation in growth rates. Building on the present model to explore how these factors can alter observable features of bacterial abundances will likely lead to further insights.

### Bacterial motility is an adaptable trait for host colonization

To further investigate a cellular mechanism to explain the immigration phenotype, we tested the capacity for motility across strains both in classical low-agar swim plate assays and by directly measuring swim velocities of individual cells. Both assays show that the evolved isolates have hypermotile phenotypes compared to the ancestor. It is unclear how this would translate into increased host immigration, but it may act by increasing the likelihood that the evolved isolate will encounter a host. Indeed, once in the host mouth, this phenotype does not promote competitive fitness, since we demonstrated that starting competitions from the host mouth (via mouth gavage) did not confer a competitive advantage (Fig 3). The importance of motility in zebrafish–microbe symbiosis has been previously reported [5,23,46]. For example, Burns and colleagues used ancestral trait reconstruction to infer that genetic pathways associated with motility and chemotaxis were enriched in microbial communities present in the guts of cohoused zebrafish (relative to those in solitary-housed zebrafish), suggesting that motility and chemotaxis may be important to dispersal-adapted gut communities [23]. Stephens and colleagues conducted a transposon-mediated mutational screen to identify genetic determinants for host colonization using two zebrafish gut isolates from different genera, *Vibrio* and *Aeromonas* (although a different species than Aer01) [5]. For both species, mutations in chemotaxis and motility genes decreased host-colonization propensity. It was not determined, however, how the impaired motility affected colonization dynamics. Rawls and colleagues previously showed that flagellar motility is important for host colonization of the zebrafish by a human opportunistic pathogen, *Pseudomonas aeruginosa*, and is required for interaction with the host immune system [46]. The role of motility in other aquatic host–microbe systems has been described as well, such as the *Vibrio*–squid symbiosis [47]. Additionally, chemotactic motility was shown to be important for maximum virulence of a pathogenic *Vibrio anguillarum* strain in rainbow trout, and this was shown to be dependent on a natural route of transmission, as motility mutants directly injected into fish did not have decreased virulence [64]. In each of these examples, the stage of colonization impacted by loss of motility functions was not fully resolved. To our knowledge, our study is the first to clearly demonstrate an association between bacterial motility and immigration into the zebrafish host.

### Bacterial adaptation to host–microbe metacommunities

This new model system was designed with the specific goal of studying bacterial host association in the context of an entire ecological framework, incorporating multiple hosts and extra- and intrahost microbial populations. Although this aquatic model system may not be an intuitive surrogate for understanding terrestrial host–microbe interactions, it is relevant to such systems in many ways. For example, like zebrafish, all animals begin life with very few to no resident microbes. Therefore, there is an initial establishment phase during which immigration of microbes into the naïve host occurs. Likewise, there is growing awareness that individual host microbiomes can be considered as part of a larger metacommunity, connected to the microbiomes of different hosts via transmission and dispersal [22,65]. Compelling evidence for this is the growing body of research showing that cohabiting animals such as mice [66,67] and humans (e.g., [58,68]) have more similar gut microbiomes than those not cohoused. Furthermore, although the mechanism of immigration may be different in different systems (e.g., swimming through the EM in our system versus transmitting between human hosts by attachment to a fomite), the trait (increased immigration) is the same. These points illustrate that the intra- and extrahost factors for host association that we consider in our model are likely broadly applicable to other systems.

We achieved the foremost goal of this study, which was to identify bacterial traits that confer host association via phenotypic characterization of the evolved isolates. Determination of the genetic basis of these adaptations would provide an even deeper understanding of the underlying biological mechanisms at play. A concession of using a mutator strain is that the increased number of mutations could make identification of adaptive ones difficult. In our evolved genomes, initial sequencing and preliminary analysis of passage 4 evolved isolates resulted in an average of 40 mutations per genome, with no obvious adaptive mutation candidates. An important next step is to repeat this study with non-mutator Aer01, in which there will be much fewer mutations, and therefore the adaptive mutations would presumably be easier to identify. This would allow more in-depth investigation of the genetic and physiological mechanisms of host association and adaptation.

Using a highly tractable model system, we were able to demonstrate that an enhanced extrahost process, immigration from the external environment, can increase host association by a bacterium. This finding challenges conventional assumptions about the primary importance of the intrahost environment in shaping host–microbe interactions. Furthermore, it supports a newly emerging paradigm that recognizes that ecological processes such as transmission may play substantive roles in microbiome assembly and dynamics.

## Materials and methods

### Ethics statement

All experiments with zebrafish were done in accordance with protocols approved by the University of Oregon Institutional Animal Care and Use Committee (IACUC); the animal protocol number for this work is 15–98 [69].

### Gnotobiotic zebrafish husbandry

All experiments involving zebrafish were conducted following standard protocols and procedures approved by the University of Oregon Institutional Care and Use Committee. For the evolution passaging and bacterial competitions, WT (AB × Tu strain) fish were used. Competition experiments were also conducted using *myd88* mutant zebrafish, which were previously generated via CRISPR-Cas9 system and verified to have the expected phenotype of an *myd88* KO mutant [23]. Fish were maintained as previously described [69]. Fish were not fed in any of the experiments described here. GF derivation of fish embryos followed protocols previously described [31]. Generally, fish were inoculated with bacterial cultures at 4 dpf. At 7 dpf, fish were euthanized with tricaine (Western Chemical), following approved procedures, and mounted in sterile 4% methylcellulose solution (Fisher), and the intestines were removed by dissection (described in [70]) and used for enumeration of colonizing bacteria or as inoculum for GF fish.

### Bacterial strains

The bacterial strain used in this study is the zebrafish isolate Aer01 (*A*. *veronii*, *PRJNA205571*) previously described [33]. Fluorescently tagged (dTomato or superfolder GFP) variants of this strain were generated using a Tn7 transposon-based system as previously described [32,34]. This method results in the integration of a cassette containing the *dTomato/GFP* gene and a gentamycin resistance gene in the chromosome at a specific target location. Subsequently, *mutS* deletion mutants (unmarked, clean deletions) were made in the fluorescently tagged genetic background strains following an allelic exchange system [34]. Strains were always grown at 30 °C, with shaking for liquid cultures.

### Fluctuation assay

The mutator phenotype of the *mutS* KO mutants was verified via fluctuation assay [71]. Briefly, overnight TSB (BD, Sparks, MD, United States) cultures of the WT, Δ*mutS*, Δ*mutS*, *attTn7*::*dTomato*, and Δ*mutS*, *attTn7*::*sfGFP* strains were diluted to 10^−7^ in TSB and then split into 2 ml aliquots in replicate tubes (WT- 20; Δ*mutS-* 20; Δ*mutS*,*attTn7*::*dTomato*- 5, and Δ*mutS*, *attTn7*::*sfGFP*—5), and cultures were grown at 30 °C with shaking overnight. Cultures were spread-plated (100 μl) on either tryptic soy agar (TSA) or TSA supplemented with 12 μg/ml rifampicin (RPI) and grew at 30 °C overnight for CFU/ml determination. Mutation rates were calculated using FALCOR (http://www.mitochondria.org/protocols/FALCOR.html [72]) with the Lea-Coulson Method of the Median (MODIFIED) option. No overt differences in fitness were detected between WT and tagged mutator strains in vitro or in vivo.

### Experiment to estimate bottleneck size of initial colonization

Overnight rich media cultures of untagged and dTomato-tagged Aer01(non-mutator) were pelleted, washed with sterile EM, and mixed (either 1:100 or 1:300, tagged:untagged). These mixed cultures were used as inoculum for flasks of (10–15) 4 dpf larval fish to a starting density of approximately 10^6^ CFU/ml. After 24-hour colonization, at 5 dpf, the fish were dissected and the guts transferred into 1.6 ml tubes containing 500 μl sterile EM and approximately 100 μl of 0.5 mm zirconium oxide beads (Next Advance, Averill Park, NY, US) and were then homogenized using a bullet blender tissue homogenizer (Next Advance, Averill Park, NY, US) for 30 seconds at power 4. One hundred microliters of the homogenate was spread-plated on TSA plates, incubated overnight, and screened by fluorescence microscopy for the presence of dTomato-tagged Aer01 colonies. The experiment was repeated twice, on independent days, with two replicate flasks for each ratio on each day. We used a binomial sampling model to estimate a bottleneck size for the first 24 hours of colonization, using the cumulative distribution function (CDF) to determine the number of cells sampled that would result in the probability of colonization observed. The CDF gives the probability of not finding a “success”—in this case, a tagged cell—given a number of trials (the number of cells sampled in 24 hours), with a given success rate (1:100 or 1:300). One minus this probability is our observed frequency of colonized fish. We can then solve for the number of trials that yield the observed colonization rate. This analysis yielded an average bottleneck estimate of 193.7 cells sampled per day, with a standard error of 140.0.

### Succession assay to determine intra- and extrahost population mixing

At 4 dpf, GF larval zebrafish were mono-associated with untagged Aer01 (non-mutator) by inoculating to approximately 10^6^ CFU/ml. At 5 dpf, dTomato-tagged Aer01 was added to the flask to the same approximately 10^6^ CFU/ml density. At 6 dpf (24 hours) and 7 dpf (48 hours), fish were dissected and the guts homogenized and plated as described above. The ratios of untagged (founder) to tagged (invader) were determined by fluorescence microscopy of the colonies.

### Fish-to-fish transmission assay

At 4 dpf, flasks of GF larval zebrafish were mono-associated with either the ancestral strain or the passage 4 (line 3) evolved isolate. At 6 dpf, two mono-associated fish (“donors”) were washed with sterile EM and then transferred into flasks containing 13 GF larval zebrafish that were 6 dpf (“recipients”). Twelve hours later, all fish in the flasks were dissected and the guts homogenized and plated to determine the colonization level of each strain in the recipient fish.

### Evolution experiment

Evolution passaging was initiated using an equal mixture of Δ*mutS*, *attTn7*::*dTomato* and Δ*mutS*, *attTn7*::*sfGFP* strains as a way of tracking gain-of-fitness lineages throughout passaging by monitoring changes in ratios of tagged populations. In all three replicate lines, the Δ*mutS*, *attTn7*::*sfGFP* populations were undetectable by passage 3, suggesting either a fitness defect in this genome or that the emergence of gain-of-fitness mutants arose in the Δ*mutS*, *attTn7*::*dTomato* lineage in all three lines by chance. The first passage was inoculated by pelleting 1 ml TSB overnight cultures of Δ*mutS*, *attTn7*::*dTomato* and Δ*mutS*, *attTn7*::*sfGFP* strains, resuspending them in 1 ml sterile EM, mixing them 1:1, and then adding them to replicate flasks of 4 dpf GF WT fish (10–15 larval fish, 15 ml EM) to a final volume of 10^6^ CFU/ml. Inoculated fish were incubated according to IACUC protocol. At 7 dpf, fish were euthanized with tricane, and the intestines were removed by dissection (described in [70]). All guts from a flask of fish were combined into a single 1.6 ml tube containing 500 μl sterile EM and approximately 100 μl of 0.5 mm zirconium oxide beads (Next Advance, Averill Park, NY, US) and were then homogenized using a bullet blender tissue homogenizer (Next Advance, Averill Park, NY, US) for 30 seconds at power 4. *Aeromonas* populations were monitored by dilution-plating a small aliquot (20 μl) of the combined gut sample and an aliquot of the flask EM on TSA plates. Half (approximately 250 μl) of the homogenate was mixed with 250 μl of sterile 50% glycerol and then stored at −80 °C. The remaining homogenate was stored at 4 °C for 4 days and then used as inocula for the subsequent flasks of 4 dpf GF fish. For subsequent inoculations, all of the 4 °C sample (about 200 μl) was added to the next flask of fish (resulting in approximately 10^4^ CFU/ml at the beginning of the passage). This process was continued for 22 passages total, for all three lines.

### Colony purification of evolved isolates

From freezer stocks of whole populations from selected evolution passages (namely, 4, 8, 14 [13, line 1], 18, and 22), cells were streaked out onto TSA plates for isolation and then incubated at 30 °C for 1 day. Isolated colonies were then picked from the plates into 5 ml TSB cultures and allowed to grow shaking at 30 °C for about 6 hours; then, 25% glycerol freezer stocks were made and stored at −80 °C.

### Bacterial competitions in vivo

For in vivo bacterial competitions, to assess fitness, selected strains were grown overnight in TSB from freezer stocks. One milliliter of the overnight cultures was pelleted (8,700 rcf, 2 minutes) and then resuspended in 1 ml sterile EM. Competing strains were mixed at about 1:10 (competitor:reference) and then added to flasks of 4 dpf GF fish (either WT or *myd88* mutant zebrafish) at about 10^6^ CFU/ml. For all competitions, ancestor (Δ*mutS*, *attTn7*::*dTomato)* or evolved isolates were competed against the WT ancestral strain, Aer01 *attTn7*::*sfGFP*. At 7 dpf, fish guts were dissected as described above, and each gut was transferred into a 1.6 ml tube containing 500 μl sterile EM and about 100 μl of bullet beads and then bullet blended as described above. Blended samples were then diluted appropriately in sterile EM, spread-plated on TSA plates, and incubated at 30 °C for 1–2 days, and the colonies were counted. Strains were differentiated by fluorescence microscopy. The limit of detection is 5 CFU/gut.

### Bacterial competitions in vitro

For in vitro competitions, selected strains were grown overnight in TSB from freezer stocks. Competing strains were mixed at about 1:4 (competitor:reference); ancestor (Δ*mutS*, *attTn7*::*dTomato)* or evolved isolates were competed against the WT ancestral strain, Aer01 *attTn7*::*sfGFP*. Mixed cultures were then used to inoculate 5 ml TSB cultures (1:100 back dilution). Cultures were incubated at 30 °C for 24 hours and then diluted and spread-plated on TSA for enumeration via fluorescence microscopy. Data are combined from three independent replicate experiments.

### Gavage experiments

Generally, the gavage protocol was followed as previously described [44], with the following modifications. Briefly, gavage needles were made by pulling 3.5-inch (Drummond #3-000-203 GIX) capillaries, microforging (DMF1000, World Precision Instruments) them to an internal diameter of approximately 30 μm, and polishing the ends. Inoculum for gavaging was prepared by pelleting 1 ml of TSB overnight cultures (8,700 rcf, 2 minutes), resuspending them in 1 ml sterile EM, and mixing competing strains at about 1:2 (competitor:reference); ancestor (Δ*mutS*, *attTn7*::*dTomato)* or evolved isolates were competed against the differentially tagged non-mutator ancestral strain, Aer01 *attTn7*::*sfGFP*. Culture mixes were then diluted 1:30 in sterile EM for gut gavage (no dilution for mouth gavage). Prepared inocula were incubated at room temperature until gavaging and flask inoculation (approximately 30–60 minutes), allowing time for acclimation to the EM. GF 5 or 6 dpf WT fish were euthanized (120 μg/ml tricaine) and transferred to 3% methylcellulose-coated gavage mold (4% agar). Culture mixes were loaded into gavage needles, and 4.6 nl of culture mix was gavaged directly into the lumen of the gut or the mouth of individual fish using a Nanoject II (Drummond Scientific Company) on the “slow” setting. After gavage, fish were rinsed in sterile EM and then transferred into sterile EM. Immediately after gavaging, flasks of GF fish were inoculated at 10^6^ CFU/ml with the same inocula used for gavaging. Approximately 5 hours post gavage, fish were euthanized and dissected, and the guts were plated as described above.

### Migration rate experiments

Selected isolates were grown overnight in TSB, at 30 °C, with shaking. Overnight cultures were diluted to 10^−4^ in EM, subcultured 1:100 in FC-EM, and grown for an additional 24 hours at 30 °C, with shaking. FC-EM was obtained by filter-sterilizing (0.2 μm) flask EM from flasks of hatched 5–6 dpf GF zebrafish larvae. Flasks of 5 or 6 dpf GF zebrafish were combined and then inoculated with FC-EM cultures to yield approximately 10^4^ CFU/ml. The fish were then split into replicate flasks containing 10 fish and 10 ml inoculated flask EM. An EM sample was taken and plated immediately to quantify CFU/ml of inoculating strain. Subsequently, about every 45 minutes (for up to about 350 minutes), all of the fish in a replicate flask were dissected and the guts transferred into 1.6 ml tubes containing 200 μl sterile EM and approximately 100 μl bullet beads. The guts were homogenized as described above and all 200 μl spread-plated on TSA plates. Flask EM samples were also plated to enumerate bacterial CFU/ml. After 24–48 hours incubation, colonies on plates were counted.

### Theoretical estimation of growth rate and colonization time

The distribution of bacterial abundances across fish allowed us to estimate the migration and growth rates of each strain. We considered a model in which migration into a fish by an individual bacterium is a stochastic event, with some probability per unit time. The statistics of the colonizers at any time are therefore governed by a Poisson distribution characterized by a mean time to entry, τ. Following entry into the host, the descendants of a colonizer obey logistic growth (initially roughly exponential, constrained by a finite carrying capacity, *K*), characterized by some growth rate *r*. For the migration rate experiments shown here, the population size is orders of magnitude less than the carrying capacity, leaving *r* as the only relevant growth parameter. For each fish gut examined by dissection and plating, we measured the bacterial abundance, *N* (CFUs). For *N* from any individual fish, one cannot separately determine τ and *r*; the same final population can be reached, for example, by rapid colonization followed by slow growth (low τ, high *r*) or by slow colonization followed by fast growth (high τ, low *r*). The distribution of *N* across multiple fish, however, constrains both τ and *r*. Roughly, high τ and high *r* gives distributions with large variances and many uncolonized fish (*N* = 0), being dominated by the stochasticity of rare migration events, whereas low τ and low *r* gives distributions with smaller variance (S11 Fig).

We fit the experimental data (i.e., each set of CFU measurements at a particular measurement time *t*) to the above model of stochastic colonization and growth, determining the best-fit parameters τ and *r*. This is done by varying τ and *r* across a range of possible values, simulating for each τ and *r* the model outcome for many (10,000) replicates, and calculating the likelihood that the simulated data match the set of measurements. This gives both the maximum-likelihood parameter values and a measure of the likelihood distribution across the full parameter range. For most datasets (41 of 50), the likelihood *p*(τ, *r* | CFU) shows a sharp peak (S12 Fig). For the remainder, it does not, indicating either limitations of this model (e.g., the neglect of exit from the gut) or experimental error or contamination; these datasets were manually discarded. The datasets from each measurement time give independent estimates of τ and *r*; the average and standard error give the final estimates of mean entry time, growth rate, and their uncertainty for each bacterial strain (Table 1).

### Swim plate assays to assess motility

Rich media swim plates were made using TSB containing 0.2% agar (VWR Life Science AMRESCO Agarose). FC-EM swim plates were made by heat-dissolving 100 mg agar in 20 ml sterile EM and then cooling slightly and adding 30 ml sterile FC-EM (0.2% agar). Media (20 ml) were added to 100 mm petri dishes, cooled for 2 hours, and then inoculated. For inoculation, TSB overnight cultures of selected strains were pelleted (8,700 rcf, 2 minutes) and resuspended in one-tenth of the culture volume, and 3 μl was used to inoculate plates. A template for inoculation was made to facilitate insulation and sampling (S6 Fig). Plates were incubated at 30 °C and then sampled and measured at 8 hours (TSB and FC-EM), 24 hours (FC-EM), and 48 hours (FC-EM). To assess motility in TSB swim plates, the diameter of the swim zone was measured in centimeters. To determine motility in FC-EM swim plates, in which cell density was too low to visualize, we sampled agar plugs from the plates at a defined distance from the point of inoculation (Fig 5) using x-tracta Gel Extractor tool (Promega, Madison, WI, US). The agar plug was transferred into a 1.6 ml tube containing 500 ml sterile EM and approximately 100 μl 0.5 mm zirconium oxide beads (Next Advance, Averill Park, NY, US), which were then homogenized using a bullet blender tissue homogenizer (Next Advance, Averill Park, NY, US) for 30 seconds at power 4. Homogenized samples were diluted and spread-plated onto TSA plates and incubated at 30 °C overnight, and colonies were counted to enumerate the number of bacterial cells that migrated to the site of sampling.

### Live imaging and measurement of cellular swim speeds

Overnight cultures (TSB) of the ancestral and evolved isolates were washed with sterile EM; then, 50 μl was added to 1 ml sterile FC-EM, and the cultures were incubated at 30 °C for approximately 4 hours to recover and acclimate. The culture was placed in a glass cuvette and imaged in a custom-built light sheet fluorescence microscope, as previously described [73]. The light sheet optically sections bulk samples; thus, the motility of the imaged bacteria is unconstrained by surfaces. Movies in a single optical plane were captured at 30 frames per second for a duration of 20 seconds, with excitation light provided by a 561 nm solid-state laser (Coherent Sapphire 20 mW; all strains express dTomato fluorescent protein). Images were analyzed using standard particle-tracking techniques, with fine localization of bacterial positions determined by a radial symmetry–based algorithm [74], the source code for which is available here: http://pages.uoregon.edu/raghu/particle_tracking.html. For each strain, four movies were recorded from each of two biologically independent replicate cultures (eight total; seven for the ancestor). An average of 6,000 (minimum of 2,300) bacteria were tracked in each movie. In Fig 5, each data point represents average bacterial swimming velocity (μm/sec) across motile cells (velocity > 5 μm/sec) in an individual movie (Fig 5E) or the fraction of the total cells that were considered motile (velocities > 5 μm/sec) in an individual movie (Fig 5F).

## Supporting information

S1 Table Results of fluctuation assay of WT and *mutS* mutant strains to estimate differences in mutation rates. WT, wild-type.
(DOCX)

S2 Table Estimates for colonization time (τ) and in vivo growth rate (*r*) for ancestral and later-evolved (passage 18) isolates. (DOCX)

S1 Data Excel spreadsheet containing data values plotted in all main and supporting figures. (XLSX)

S1 Fig Aer01 colonization is robust over a range of inoculum concentrations. (A) CFU/gut at 48 hours post inoculation. Each point represents a single fish. Bars represent median and interquartile range. (B) CFU/ml of Aer01 in the aquatic environment of the flasks from panel A, throughout the colonization. Underlying data for A–B are provided in S1 Data. CFU/gut, colony-forming unit per gut.
(TIF)

S2 Fig Investigation of cumulative bottleneck size for the initial 24-hour Aer01 colonization of the larval zebrafish. Larval fish were inoculated with WT and *attTn7*::*dTomato* Aer01, mixed 1:100 and 1:300 (WT: *attTn7*::*dTomato)*. After a 24-hour colonization, the fish were dissected and the guts plated to determine if the minority strain (*attTn7*::*dTomato)* was present. The proportion of fish with detected or undetected *attTn7*::*dTomato* is presented. WT, wild type.
(TIF)

S3 Fig Determination of intra- and extrahost population mixing in established gut communities. Fish were mono-associated with dTomato-tagged Aer01 (primary strain) for 24 hours, inoculated with untagged Aer01 (secondary strain), and dissected 24 and 48 hours later to determine the ratio of primary:secondary strains in the guts. Each point represents an individual fish. Bars represent median and interquartile ranges. Underlying data are provided in S1 Data.
(TIF)

S4 Fig Aer01 colonization is stable across host development. GF larval zebrafish were inoculated at 4, 5, or 6 dpf and then dissected at 24 hours post inoculation, and the intestines were plated to determine CFU/gut. Bars represent the median and interquartile ranges. Underlying data are provided in S1 Data. CFU/gut, colony-forming unit per gut; dpf, days post fertilization; GF, germ-free.
(TIF)

S5 Fig Evolved isolates do not have a competitive advantage in vitro in rich media. CIs of evolved isolates and the ancestor when competed against a differentially tagged ancestral strain in vitro. Each circle represents a CI from an independent biological replicate. Underlying data are provided in S1 Data. CI, competitive index.
(TIF)

S6 Fig EM abundance of strains in the migration rate experiments. Data combined from three (ancestor), two (evolved, line 1), or one (evolved, lines 2 and 3) independent experiments; means (± SEM) are plotted for ancestor and line 1. Underlying data are provided in S1 Data. EM, embryo medium.
(TIFF)

S7 Fig Passage 4 (line 3) evolved isolate demonstrates increased fish-to-fish transmission compared to the ancestor. Mono-associated “donor” fish were incubated with 13 GF “recipient” fish for 12 hours, the guts were dissected, and the abundance of Aer01 in the fish was determined. *p*-Value = 0.003, determined using two-tailed Student’s *t* test, excluding the donor fish points. Underlying data are provided in S1 Data. GF, germ-free.
(TIF)

S8 Fig Further-evolved (passage 18) isolates demonstrate increased host immigration. (A) Groups of mono-associated fish were dissected and plated about every 45 minutes, and the fraction of colonized hosts was determined. A higher proportion of fish are colonized at earlier time points for the evolved isolates compared to the ancestor. Mean (± SEM) is plotted for ancestor; ancestor data are the same as those plotted in Fig 4A. (B) CFU/gut (mean ± SEM) from the same samples presented in A show higher gut abundance for the evolved isolates. Ancestor data are the same as those plotted in Fig 4B. (C) EM abundance (CFU/ml) shows strains do not have differences in survival in flask EM. Mean (± SEM) is plotted for ancestor. Data combined from three (ancestor) or one (evolved) independent experiment. Underlying data for A–C are provided in S1 Data. CFU/gut, colony-forming unit per gut; EM, embryo medium.
(TIFF)

S9 Fig Ancestral Aer01 has equal fitness when competed against a differentially tagged ancestral strain in either WT (circles) or *myd88*^*—*^(squares) larval zebrafish. CI = (ancestor,evolved/ref)_end_/(ancestor,evolved/ref)_start._ Each data point represents a CI from an individual fish. Line = median. Underlying data are provided in S1 Data. CI, competitive index; WT, wild-type.
(TIF)

S10 Fig Line 3, passage 4 isolate demonstrates increased host immigration in *myd88*^*-*^ hosts. A higher proportion of fish are colonized at earlier time points for the evolved isolate compared to the ancestor. Mean (± SEM) is plotted for ancestor. Data combined from three (ancestor) or one (evolved) independent experiment. Ancestor data are the same as those plotted in Fig 4A. Underlying data are provided in S1 Data.
(TIF)

S11 Fig Simulated output from a stochastic colonization and growth model. Histograms of bacterial population (*N*) across 10,000 fish at *t* = 120 minutes, with two different parameter values characterizing the migration and growth rates, each of which give the same mean population (*N* = 12) but show very different distributions. Note the large fraction of uncolonized fish (*N* = 0) if the mean entry time (τ) is large. Underlying data are provided in S1 Data.
(TIF)

S12 Fig Probability *p*(τ, *r* | CFU) of the colonization and growth model matching measured CFU values for the ancestral strain, measured at t = 135 minutes, showing a sharp peak at a particular set of parameter values. Underlying data are provided in S1 Data. CFU, colony-forming unit.
(TIF)

## Abbreviations

CFU/gut: colony-forming units per gut
CI: competitive index
dpf: days post fertilization
EM: embryo medium
FC-EM: fish-conditioned embryo medium
GF: germ-free
GFP: green fluorescent protein
TSB: tryptic soy broth
WT: wild-type

## References

[1] McFall-Ngai M, Hadfield MG, Bosch TCG, Carey HV, Domazet-Lošo T, Douglas AE, et al Animals in a bacterial world, a new imperative for the life sciences. Proceedings of the National Academy of Sciences. National Acad Sciences; 2013 2 26;110(9):3229–36. 10.1073/pnas.1218525110 PMC3587249 23391737

[2] Sironi M, Cagliani R, Forni D, Clerici M. Evolutionary insights into host-pathogen interactions from mammalian sequence data. Nature Reviews Genetics. 2015 4;16(4):224–36. 10.1038/nrg3905 25783448 PMC7096838

[3] Lynch SV, Pedersen O. The Human Intestinal Microbiome in Health and Disease. N Engl J Med. 2016 12 15;375(24):2369–79. 10.1056/NEJMra1600266 27974040

[4] Thompson LR, Sanders JG, McDonald D, Amir A, Ladau J, Locey KJ, et al A communal catalogue reveals Earth’s multiscale microbial diversity. Nature. Nature Publishing Group; 2017 11 23;551(7681):457–63. 10.1038/nature24621 PMC6192678 29088705

[5] Stephens WZ, Wiles TJ, Martinez ES, Jemielita M, Burns AR, Parthasarathy R, et al Identification of Population Bottlenecks and Colonization Factors during Assembly of Bacterial Communities within the Zebrafish Intestine. MBio. American Society for Microbiology; 2015;6(6):e01163–15. 10.1128/mBio.01163-15 PMC4626852 26507229

[6] Lee SM, Donaldson GP, Mikulski Z, Boyajian S, Ley K, Mazmanian SK. Bacterial colonization factors control specificity and stability of the gut microbiota. Nature. 2013 9 19;501(7467):426–9. 10.1038/nature12447 23955152 PMC3893107

[7] Goodman AL, McNulty NP, Zhao Y, Leip D, Mitra RD, Lozupone CA, et al Identifying genetic determinants needed to establish a human gut symbiont in its habitat. Cell Host & Microbe. 2009 9 17;6(3):279–89. 19748469 10.1016/j.chom.2009.08.003 PMC2895552

[8] Fu Y, Waldor MK, Mekalanos JJ. Tn-Seq analysis of Vibrio cholerae intestinal colonization reveals a role for T6SS-mediated antibacterial activity in the host. Cell Host & Microbe. Elsevier; 2013 12 11;14(6):652–63. 10.1016/j.chom.2013.11.001 PMC3951154 24331463

[9] Xu Q, Dziejman M, Mekalanos JJ. Determination of the transcriptome of Vibrio cholerae during intraintestinal growth and midexponential phase in vitro. Proc Natl Acad Sci USA. National Acad Sciences; 2003 2 4;100(3):1286–91. 10.1073/pnas.0337479100 PMC298765 12552086

[10] Licandro-Seraut H, Scornec H, Pédron T, Cavin J-F, Sansonetti PJ. Functional genomics of Lactobacillus casei establishment in the gut. Proceedings of the National Academy of Sciences. 2014 7 29;111(30):E3101–9. 10.1073/pnas.1411883111 PMC4121828 25024222

[11] Xu J, Mahowald MA, Ley RE, Lozupone CA, Hamady M, Martens EC, et al Evolution of Symbiotic Bacteria in the Distal Human Intestine. PLoS Biol. 2007;5(7):e156 10.1371/journal.pbio.0050156 17579514 PMC1892571

[12] Lee J-Y, Han GG, Kim EB, Choi Y-J. Comparative genomics of Lactobacillus salivarius strains focusing on their host adaptation. Microbiol Res. 2017 12;205:48–58. 10.1016/j.micres.2017.08.008 28942844

[13] Frese SA, Benson AK, Tannock GW, Loach DM, Kim J, Zhang M, et al The Evolution of Host Specialization in the Vertebrate Gut Symbiont Lactobacillus reuteri. Guttman DS, editor. PLoS Genet. 2011 2 17;7(2):e1001314 10.1371/journal.pgen.1001314 21379339 PMC3040671

[14] Kawecki TJ, Lenski RE, Ebert D, Hollis B, Olivieri I, Whitlock MC. Experimental evolution. Trends Ecol Evol (Amst). 2012 10;27(10):547–60. 22819306 10.1016/j.tree.2012.06.001

[15] Adams J, Rosenzweig F. Experimental microbial evolution: history and conceptual underpinnings. Genomics. 2014 12;104(6 Pt A):393–8. 10.1016/j.ygeno.2014.10.004 25315137

[16] Hoang KL, Morran LT, Gerardo NM. Experimental Evolution as an Underutilized Tool for Studying Beneficial Animal–Microbe Interactions. Front Microbiol. Frontiers; 2016 9 13;07(e1004182):109–16. 10.3389/fmicb.2016.01444 PMC5020044 27679620

[17] Kohl KD, Sadowska ET, Rudolf AM, Dearing MD, Koteja P. Experimental Evolution on a Wild Mammal Species Results in Modifications of Gut Microbial Communities. Front Microbiol. 2016;7:634 10.3389/fmicb.2016.00634 27199960 PMC4854874

[18] Giraud A, Matic I, Tenaillon O, Clara A, Radman M, Fons M, et al Costs and Benefits of High Mutation Rates: Adaptive Evolution of Bacteria in the Mouse Gut. Science. American Association for the Advancement of Science; 2001 3 30;291(5513):2606–8. 10.1126/science.1056421 11283373

[19] Giraud A, Arous S, De Paepe M, Gaboriau-Routhiau V, Bambou J-C, Rakotobe S, et al Dissecting the genetic components of adaptation of Escherichia coli to the mouse gut. PLoS Genet. 2008 1;4(1):e2 10.1371/journal.pgen.0040002 18193944 PMC2174974

[20] Barroso-Batista J, Sousa A, Lourenço M, Bergman M-L, Sobral D, Demengeot J, et al The First Steps of Adaptation of Escherichia coli to the Gut Are Dominated by Soft Sweeps. Coop G, editor. PLoS Genet. 2014; 10(3):e1004182 10.1371/journal.pgen.1004182 24603313 PMC3945185

[21] Jones MA, Marston KL, Woodall CA, Maskell DJ, Linton D, Karlyshev AV, et al Adaptation of Campylobacter jejuni NCTC11168 to high-level colonization of the avian gastrointestinal tract. Infection and Immunity. American Society for Microbiology; 2004 7;72(7):3769–76. 10.1128/IAI.72.7.3769-3776.2004 PMC427441 15213117

[22] Adair KL, Douglas AE. ScienceDirect Making a microbiome: the many determinants of host-associated microbial community composition. Current Opinion in Microbiology. Elsevier Ltd; 2017 2 1;35:23–9. 10.1016/j.mib.2016.11.002 27907842

[23] Burns AR, Miller E, Agarwal M, Rolig AS, Milligan-Myhre K, Seredick S, et al Interhost dispersal alters microbiome assembly and can overwhelm host innate immunity in an experimental zebrafish model. Proc Natl Acad Sci USA. 2017 10 2;8:201702511–6. 10.1073/pnas.1702511114 PMC5651736 28973938

[24] Burns AR, Stephens WZ, Stagaman K, Wong S, Rawls JF, Guillemin K, et al Contribution of neutral processes to the assembly of gut microbial communities in the zebrafish over host development. ISME J. 2016 3;10(3):655–64. 10.1038/ismej.2015.142 26296066 PMC4817674

[25] Browne HP, Neville BA, Forster SC, Lawley TD. Transmission of the gut microbiota: spreading of health. Nature Publishing Group. Nature Publishing Group; 2017 9;15(9):531–43. 10.1038/nrmicro.2017.50 PMC5837012 28603278

[26] Tung J, Barreiro LB, Burns MB, Grenier J-C, Lynch J, Grieneisen LE, et al Social networks predict gut microbiome composition in wild baboons. eLife. eLife Sciences Publications Limited; 2015 3 16;4:e05224. 10.7554/eLife.05224 PMC4379495 25774601

[27] Gilbert SF. A holobiont birth narrative: the epigenetic transmission of the human microbiome. Front Genet. Frontiers; 2014;5:282. 10.3389/fgene.2014.00282 PMC4137224 25191338

[28] Storelli G, Strigini M, Grenier T, Bozonnet L, Schwarzer M, Daniel C, et al Drosophila Perpetuates Nutritional Mutualism by Promoting the Fitness of Its Intestinal Symbiont Lactobacillus plantarum. Cell Metabolism. Elsevier Inc; 2018 2 6;27(2):362–8. 10.1016/j.cmet.2017.11.011 PMC5807057 29290388

[29] Martino ME, Joncour P, Leenay R, Gervais H, Shah M, Hughes S, et al Bacterial Adaptation to the Host’s Diet Is a Key Evolutionary Force Shaping Drosophila-Lactobacillus Symbiosis. Cell Host & Microbe. 2018 7 11;24(1):109–119.e6. 30008290 10.1016/j.chom.2018.06.001 PMC6054917

[30] Burns AR, Guillemin K. The scales of the zebrafish: host-microbiota interactions from proteins to populations. Current Opinion in Microbiology. 2017 8;38:137–41. 10.1016/j.mib.2017.05.011 28618368 PMC5705389

[31] Melancon E, Gomez De La Torre Canny S, Sichel S, Kelly M, Wiles TJ, Rawls JF, et al Best practices for germ-free derivation and gnotobiotic zebrafish husbandry. Methods Cell Biol. Elsevier; 2017;138:61–100. 10.1016/bs.mcb.2016.11.005 PMC5568843 28129860

[32] Wiles TJ, Jemielita M, Baker RP, Schlomann BH, Logan SL, Ganz J, et al Host Gut Motility Promotes Competitive Exclusion within a Model Intestinal Microbiota. PLoS Biol. Public Library of Science; 2016 7;14(7):e1002517. 10.1371/journal.pbio.1002517 PMC4961409 27458727

[33] Stephens WZ, Burns AR, Stagaman K, Wong S, Rawls JF, Guillemin K, et al The composition of the zebrafish intestinal microbial community varies across development. ISME J. 2016 3;10(3):644–54. 10.1038/ismej.2015.140 26339860 PMC4817687

[34] Wiles TJ, Wall ES, Schlomann BH, Hay EA, Parthasarathy R, Guillemin K. Modernized Tools for Streamlined Genetic Manipulation and Comparative Study of Wild and Diverse Proteobacterial Lineages. Ruby EG, editor. MBio. American Society for Microbiology; 2018 10 9;9(5):e01877–18. 10.1128/mBio.01877-18 PMC6178617 30301859

[35] Bates JM, Mittge E, Kuhlman J, Baden KN, Cheesman SE, Guillemin K. Distinct signals from the microbiota promote different aspects of zebrafish gut differentiation. Dev Biol. 2006 9 15;297(2):374–86. 10.1016/j.ydbio.2006.05.006 16781702

[36] Cheesman SE, Neal JT, Mittge E, Seredick BM, Guillemin K. Epithelial cell proliferation in the developing zebrafish intestine is regulated by the Wnt pathway and microbial signaling via Myd88. Proceedings of the National Academy of Sciences. 2011 3 15;108 Suppl 1:4570–7. 10.1073/pnas.1000072107 PMC3063593 20921418

[37] Hill JH, Franzosa EA, Huttenhower C, Guillemin K. A conserved bacterial protein induces pancreatic beta cell expansion during zebrafish development. eLife. 2016 12 13;5 10.7554/eLife.20145 27960075 PMC5154760

[38] Rolig AS, Parthasarathy R, Burns AR, Bohannan BJM, Guillemin K. Individual Members of the Microbiota Disproportionately Modulate Host Innate Immune Responses. Cell Host & Microbe. 2015 11 11;18(5):613–20. 26567512 10.1016/j.chom.2015.10.009 PMC4701053

[39] Denamur E, Matic I. Evolution of mutation rates in bacteria. Molecular Microbiology. Blackwell Publishing Ltd; 2006 5 1;60(4):820–7. 10.1111/j.1365-2958.2006.05150.x 16677295

[40] Shaver AC, Dombrowski PG, Sweeney JY, Treis T, Zappala RM, Sniegowski PD. Fitness evolution and the rise of mutator alleles in experimental Escherichia coli populations. Genetics. Genetics Society of America; 2002 10;162(2):557–66. 10.1093/genetics/162.2.557 PMC1462288 12399371

[41] Wong A, Rodrigue N, Kassen R. Genomics of Adaptation during Experimental Evolution of the Opportunistic Pathogen Pseudomonas aeruginosa. PLoS Genet. 2012;8(9):e1002928 10.1371/journal.pgen.1002928 23028345 PMC3441735

[42] Sokurenko E. Pathoadaptive Mutations in Uropathogenic Escherichia coli. Microbiol Spectr. 2016 4;4(2). 10.1128/microbiolspec.UTI-0020-2015 27227300

[43] Ciofu O, Mandsberg LF, Bjarnsholt T, Wassermann T, Høiby N. Genetic adaptation of Pseudomonas aeruginosa during chronic lung infection of patients with cystic fibrosis: strong and weak mutators with heterogeneous genetic backgrounds emerge in mucA and/or lasR mutants. Microbiology (Reading, Engl). Society for General Microbiology; 2010 4 1;156(4):1108–19. 10.1099/mic.0.033993-0 20019078

[44] Cocchiaro JL, Rawls JF. Microgavage of zebrafish larvae. J Vis Exp. 2013 2 20;(72):e4434 10.3791/4434 23463135 PMC3605733

[45] Ottemann KM, Miller JF. Roles for motility in bacterial-host interactions. Molecular Microbiology. 1997 6;24(6):1109–17. 9218761 10.1046/j.1365-2958.1997.4281787.x

[46] Rawls JF, Mahowald MA, Goodman AL, Trent CM, Gordon JI. In vivo imaging and genetic analysis link bacterial motility and symbiosis in the zebrafish gut. Proc Natl Acad Sci USA. 2007 5 1;104(18):7622–7. 10.1073/pnas.0702386104 17456593 PMC1855277

[47] Brennan CA, Mandel MJ, Gyllborg MC, Thomasgard KA, Ruby EG. Genetic determinants of swimming motility in the squid light-organ symbiont Vibrio fischeri. Microbiologyopen. 2013 8;2(4):576–94. 10.1002/mbo3.96 23907990 PMC3948606

[48] Millikan DS, Ruby EG. Alterations in Vibrio fischeri motility correlate with a delay in symbiosis initiation and are associated with additional symbiotic colonization defects. Appl Microbiol. 2002 5;68(5):2519–28. 10.1128/AEM.68.5.2519-2528.2002 PMC127559 11976129

[49] Josenhans C, Suerbaum S. The role of motility as a virulence factor in bacteria. Int J Med Microbiol. 2002 3;291(8):605–14. 10.1078/1438-4221-00173 12008914

[50] Chaban B, Hughes HV, Beeby M. The flagellum in bacterial pathogens: For motility and a whole lot more. Semin Cell Dev Biol. 2015 10;46:91–103. 10.1016/j.semcdb.2015.10.032 26541483

[51] Bates JM, Akerlund J, Mittge E, Guillemin K. Intestinal alkaline phosphatase detoxifies lipopolysaccharide and prevents inflammation in zebrafish in response to the gut microbiota. Cell Host & Microbe. 2007 12 13;2(6):371–82. 18078689 10.1016/j.chom.2007.10.010 PMC2730374

[52] Hooper LV. Do symbiotic bacteria subvert host immunity? Nat Rev Micro. 2009 5;7(5):367–74. 10.1038/nrmicro2114 19369952

[53] van der Vaart M, van Soest JJ, Spaink HP, Meijer AH. Functional analysis of a zebrafish myd88 mutant identifies key transcriptional components of the innate immune system. Dis Model Mech. 2013 4 24;6(3):841–54. 10.1242/dmm.010843 23471913 PMC3634667

[54] Troll JV, Hamilton MK, Abel ML, Ganz J, Bates JM, Stephens WZ, et al Microbiota promote secretory cell determination in the intestinal epithelium by modulating host Notch signaling. Development. 2018 2 23;145(4). 10.1242/dev.155317 PMC5869004 29475973

[55] Oh J, Byrd AL, Park M, NISC Comparative Sequencing Program, Kong HH, Segre JA. Temporal Stability of the Human Skin Microbiome. Cell. 2016 5 5;165(4):854–66. 10.1016/j.cell.2016.04.008 27153496 PMC4860256

[56] Rajilić-Stojanović M, Heilig HGHJ, Tims S, Zoetendal EG, de Vos WM. Long-term monitoring of the human intestinal microbiota composition. Environ Microbiol. 2013 4 4;15(4):1146–59. 10.1111/1462-2920.12023 23286720

[57] Linnenbrink M, Wang J, Hardouin EA, Künzel S, Metzler D, Baines JF. The role of biogeography in shaping diversity of the intestinal microbiota in house mice. Mol Ecol. 2013 4;22(7):1904–16. 10.1111/mec.12206 23398547

[58] Song SJ, Lauber C, Costello EK, Lozupone CA, Humphrey G, Berg-Lyons D, et al Cohabiting family members share microbiota with one another and with their dogs. eLife. 2013 4 16;2:6378–22. 10.7554/eLife.00458 PMC3628085 23599893

[59] Martínez I, Stegen JC, Maldonado-Gómez MX, Eren AM, Siba PM, Greenhill AR, et al The gut microbiota of rural papua new guineans: composition, diversity patterns, and ecological processes. Cell Rep. 2015 4 28;11(4):527–38. 10.1016/j.celrep.2015.03.049 25892234

[60] Stagaman K, Burns AR, Guillemin K, Bohannan BJ. The role of adaptive immunity as an ecological filter on the gut microbiota in zebrafish. ISME J. Nature Publishing Group; 2017 7;11(7):1630–9. 10.1038/ismej.2017.28 PMC5520148 28304369

[61] Alizon S, Hurford A, Mideo N, Van Baalen M. Virulence evolution and the trade-off hypothesis: history, current state of affairs and the future. J Evolution Biol. 2009 2;22(2):245–59. 10.1111/j.1420-9101.2008.01658.x 19196383

[62] Ewald PW. Transmission modes and the evolution of virulence: With special reference to cholera, influenza, and AIDS. Hum Nat. 2nd ed. 1991 3;2(1):1–30. 10.1007/BF02692179 24222188

[63] Schlomann BH. Stationary moments, diffusion limits, and extinction times for logistic growth with random catastrophes. Journal of Theoretical Biology. 2018 10 7;454:154–63. 10.1016/j.jtbi.2018.06.007 29885410 PMC6089642

[64] O’Toole R, Milton DL, Wolf-Watz H. Chemotactic motility is required for invasion of the host by the fish pathogen Vibrio anguillarum. Molecular Microbiology. 1996 2;19(3):625–37. 8830252 10.1046/j.1365-2958.1996.412927.x

[65] Costello EK, Stagaman K, Dethlefsen L, Bohannan BJM, Relman DA. The Application of Ecological Theory Toward an Understanding of the Human Microbiome. Science. 2012 6 7;336(6086):1255–62. 10.1126/science.1224203 22674335 PMC4208626

[66] Hildebrand F, Nguyen TLA, Brinkman B, Yunta RG, Cauwe B, Vandenabeele P, et al Inflammation-associated enterotypes, host genotype, cage and inter-individual effects drive gut microbiota variation in common laboratory mice. Genome Biol. 2013 1 24;14(1):R4 10.1186/gb-2013-14-1-r4 23347395 PMC4053703

[67] McCafferty J, Mühlbauer M, Gharaibeh RZ, Arthur JC, Perez-Chanona E, Sha W, et al Stochastic changes over time and not founder effects drive cage effects in microbial community assembly in a mouse model. ISME J. Nature Publishing Group; 2013 11 1;7(11):2116–25. 10.1038/ismej.2013.106 PMC3806260 23823492

[68] Turroni S, Rampelli S, Biagi E, Consolandi C, Severgnini M, Peano C, et al Temporal dynamics of the gut microbiota in people sharing a confined environment, a 520-day ground-based space simulation, MARS500. Microbiome. BioMed Central; 2017 12 1;5(1):39. 10.1186/s40168-017-0256-8 PMC5366131 28340597

[69] Westerfield M. The zebrafish book A guide for the laboratory use of zebrafish (Danio rerio). Eugene: University of Oregon; 2000.

[70] Milligan-Myhre K, Charette JR, Phennicie RT, Stephens WZ, Rawls JF, Guillemin K, et al Study of Host–Microbe Interactions in Zebrafish Third Edition Vol. 105, Methods in Cell Biology. Elsevier Inc; 2011 30 p. 10.1016/B978-0-12-381320-6.00004-7 PMC4700925 21951527

[71] Crane G. A modified Luria-Delbrück fluctuation assay for estimating and comparing mutation rates. Mutation Research/Fundamental and Molecular Mechanisms of Mutagenesis. 1996 7 22;354(2):171–82. 8764946 10.1016/0027-5107(96)00009-7

[72] Hall BM, Ma C-X, Liang P, Singh KK. Fluctuation analysis CalculatOR: a web tool for the determination of mutation rate using Luria-Delbruck fluctuation analysis. Bioinformatics. 2009 6 15;25(12):1564–5. 10.1093/bioinformatics/btp253 19369502 PMC2687991

[73] Taormina MJ, Jemielita M, Stephens WZ, Burns AR, Troll JV, Parthasarathy R, et al Investigating bacterial-animal symbioses with light sheet microscopy. The Biological Bulletin. MBL; 2012;223(1):7–20. 10.1086/BBLv223n1p7 PMC3952068 22983029

[74] Parthasarathy R. Rapid, accurate particle tracking by calculation of radial symmetry centers. Nature Methods. 2012 6 10;9(7):724–6. 10.1038/nmeth.2071 22688415

