# Supplementary material for: Experimental bacterial adaptation to the zebrafish gut reveals a primary role for immigration
Source: PLoS Biol. 2018 Dec 10;16(12):e2006893. doi: 10.1371/journal.pbio.2006893 (PMC6301714; doi:10.1371/journal.pbio.2006893)
Supplement: S1 Table — WT, wild-type. (DOCX) [file pbio.2006893.s001.docx]

| **Strain** | **Rif^R^ mutants/culture (median)** | **Mutation Rate** |
| --- | --- | --- |
| WT | 2.30 x 10^2^ | 8.30 x 10^-10^ |
| *ΔmutS* | 2.58 x 10^5^ | 1.17 x 10^-6^ |
| *ΔmutS (GFP-tagged* | 1.70 x 10^5^ | 1.51 x 10^-6^ |
| *ΔmutS (dTomato-tagged)* | 1.25 x 10^5^ | 1.14 x 10^-6^ |

**S1 Table.** Results of fluctuation assay of WT and *mutS* mutant strains to estimate differences in mutation rates.
