# Supplementary material for: Experimental bacterial adaptation to the zebrafish gut reveals a primary role for immigration
Source: PLoS Biol. 2018 Dec 10;16(12):e2006893. doi: 10.1371/journal.pbio.2006893 (PMC6301714; doi:10.1371/journal.pbio.2006893)
Supplement: S2 Table — (DOCX) [file pbio.2006893.s002.docx]

**S2 Table**. Estimates for colonization time (τ) and *in vivo* growth rate (r) for ancestral and later-evolved (passage 18) isolates.

| **Strain** | **τ (SE), min** | ***r* (SE),**  **1/min** | **N** |
| --- | --- | --- | --- |
| Ancestor | 130.0 (27.9) | 0.0240 (0.0063) | 9 |
| Evolved, Line 1 | 85.0 (20.9) | 0.0197 (0.0035) | 6 |
| Evolved, Line 2 | 45.0 (7.6) | 0.0207 (0.0036) | 6 |

N- number of time points combined for determination of mean and standard error
