# Supplementary figures and images for: Experimental bacterial adaptation to the zebrafish gut reveals a primary role for immigration
Source: PLoS Biol. 2018 Dec 10;16(12):e2006893. doi: 10.1371/journal.pbio.2006893 (PMC6301714; doi:10.1371/journal.pbio.2006893)

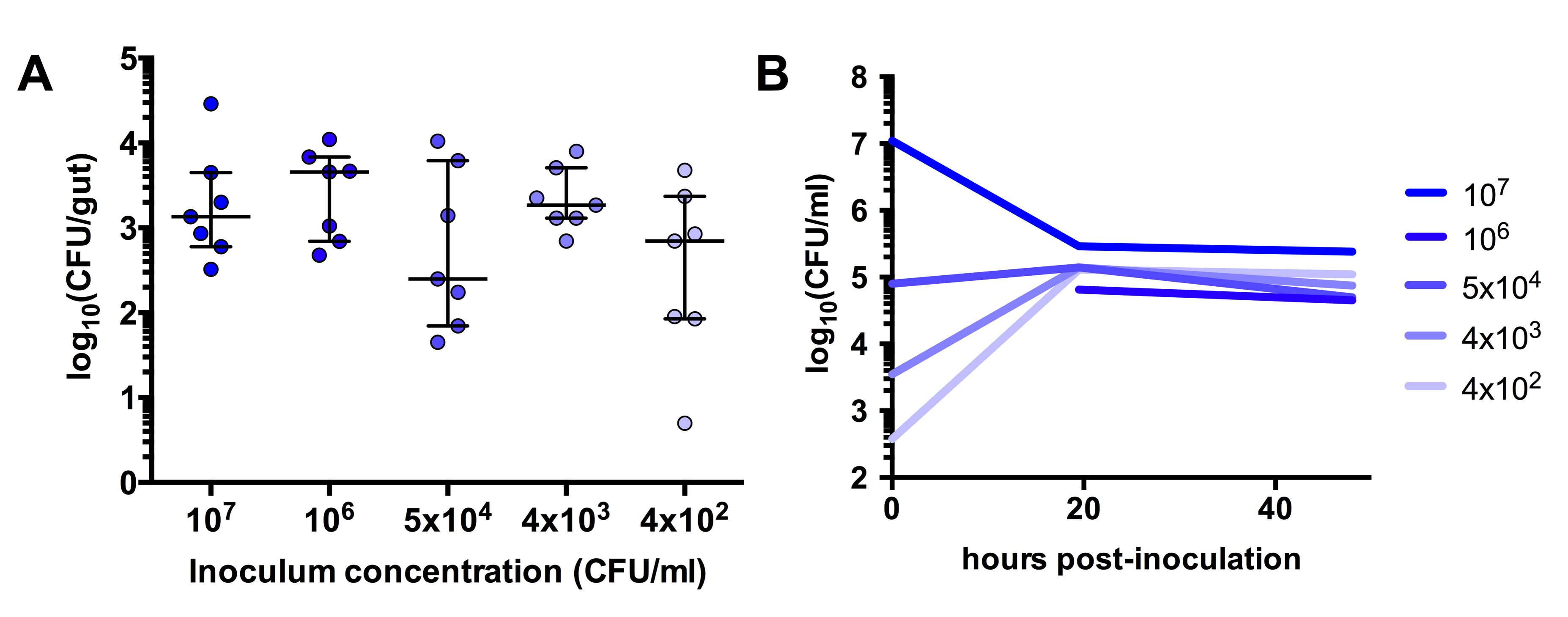

Supplement: S1 Fig — (A) CFU/gut at 48 hours post inoculation. Each point represents a single fish. Bars represent median and interquartile range. (B) CFU/ml of Aer01 in the aquatic environment of the flasks from panel A, throughout the colonization. Underlying data for A–B are provided in S1 Data. CFU/gut, colony-forming unit per gut. (TIF) [file pbio.2006893.s004.tif]

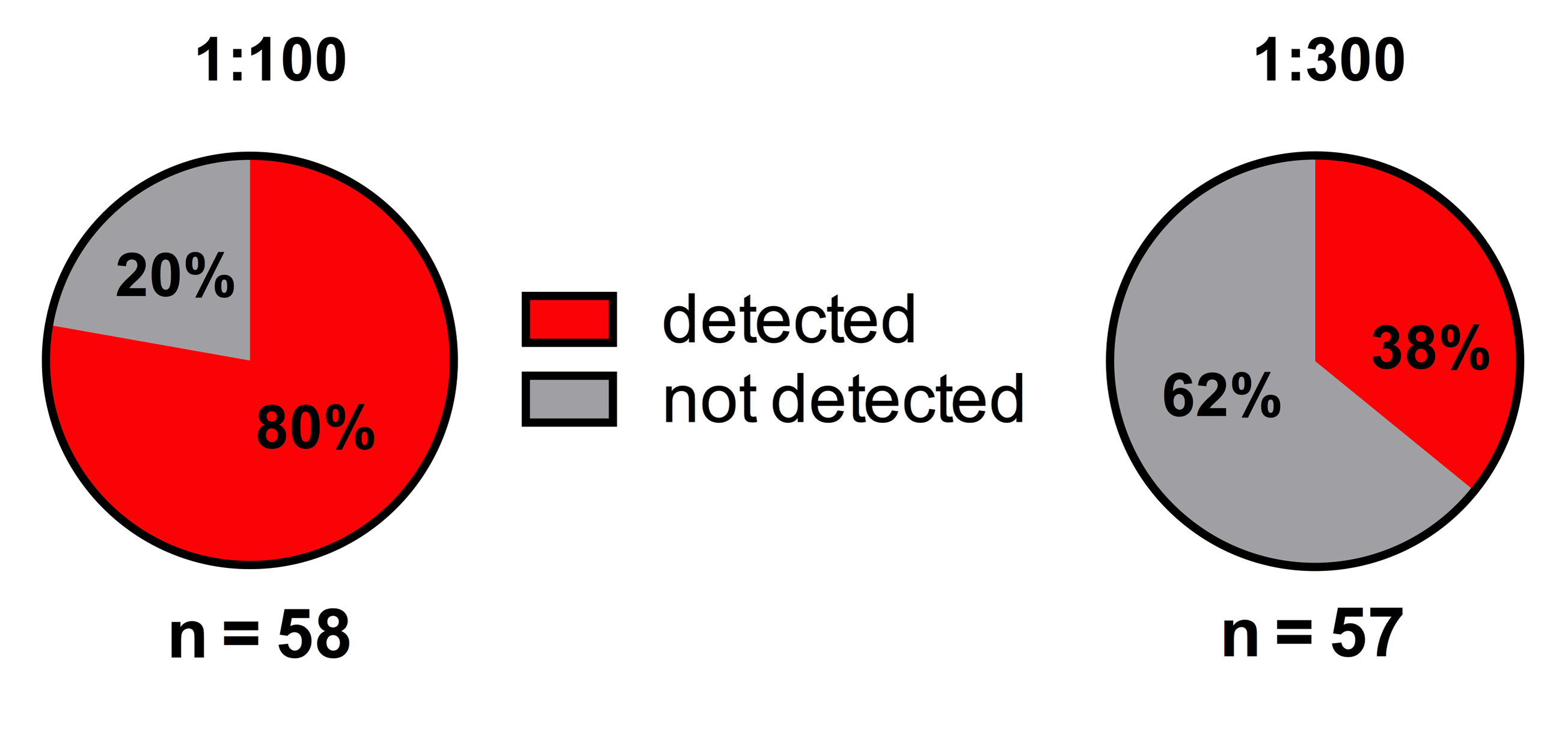

Supplement: S2 Fig — Larval fish were inoculated with WT and attTn7::dTomato Aer01, mixed 1:100 and 1:300 (WT: attTn7::dTomato). After a 24-hour colonization, the fish were dissected and the guts plated to determine if the minority strain (attTn7::dTomato) was present. The proportion of fish with detected or undetected attTn7::dTomato is presented. WT, wild type. (TIF) [file pbio.2006893.s005.tif]

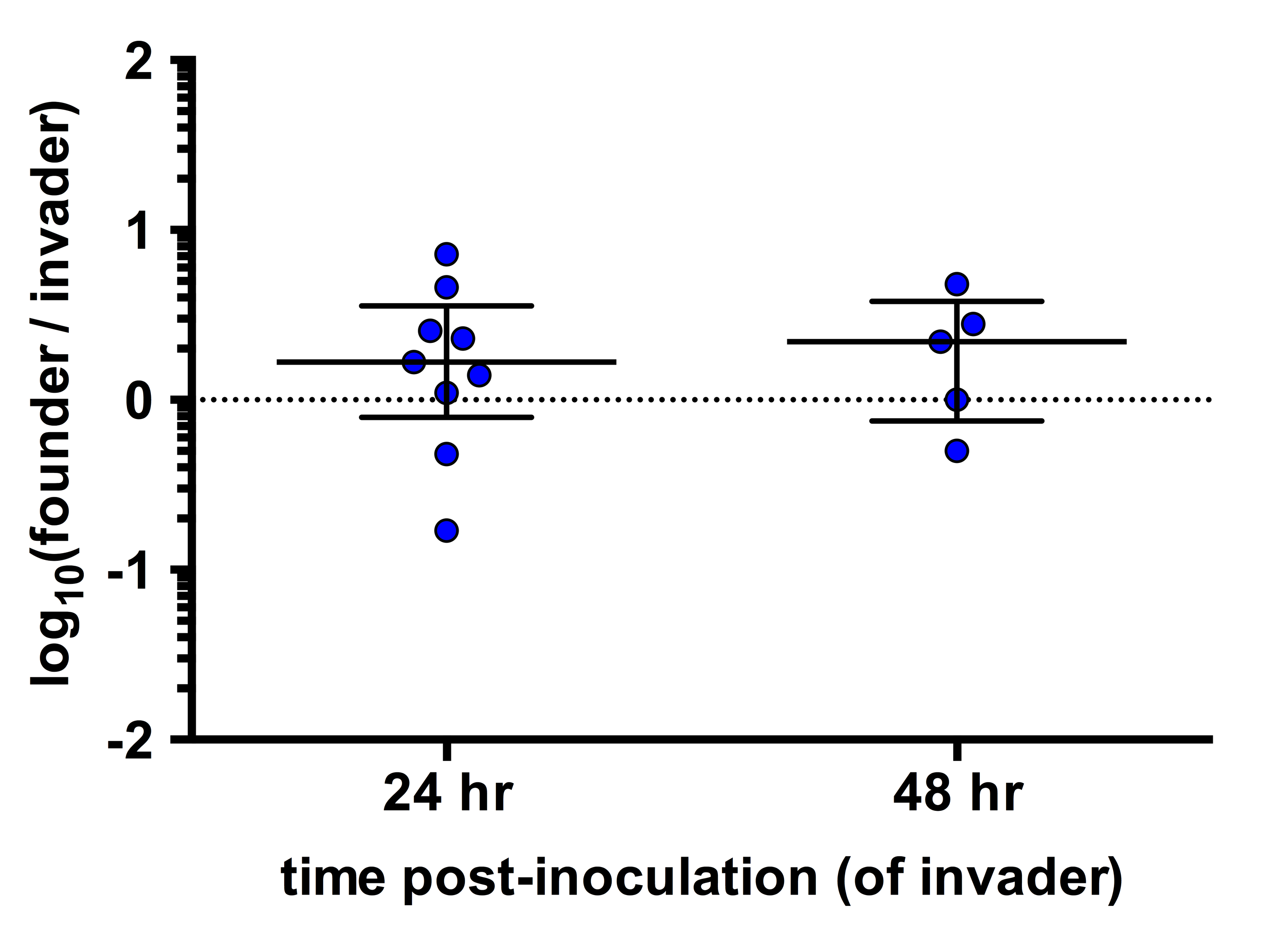

Supplement: S3 Fig — Fish were mono-associated with dTomato-tagged Aer01 (primary strain) for 24 hours, inoculated with untagged Aer01 (secondary strain), and dissected 24 and 48 hours later to determine the ratio of primary:secondary strains in the guts. Each point represents an individual fish. Bars represent median and interquartile ranges. Underlying data are provided in S1 Data. (TIF) [file pbio.2006893.s006.tif]

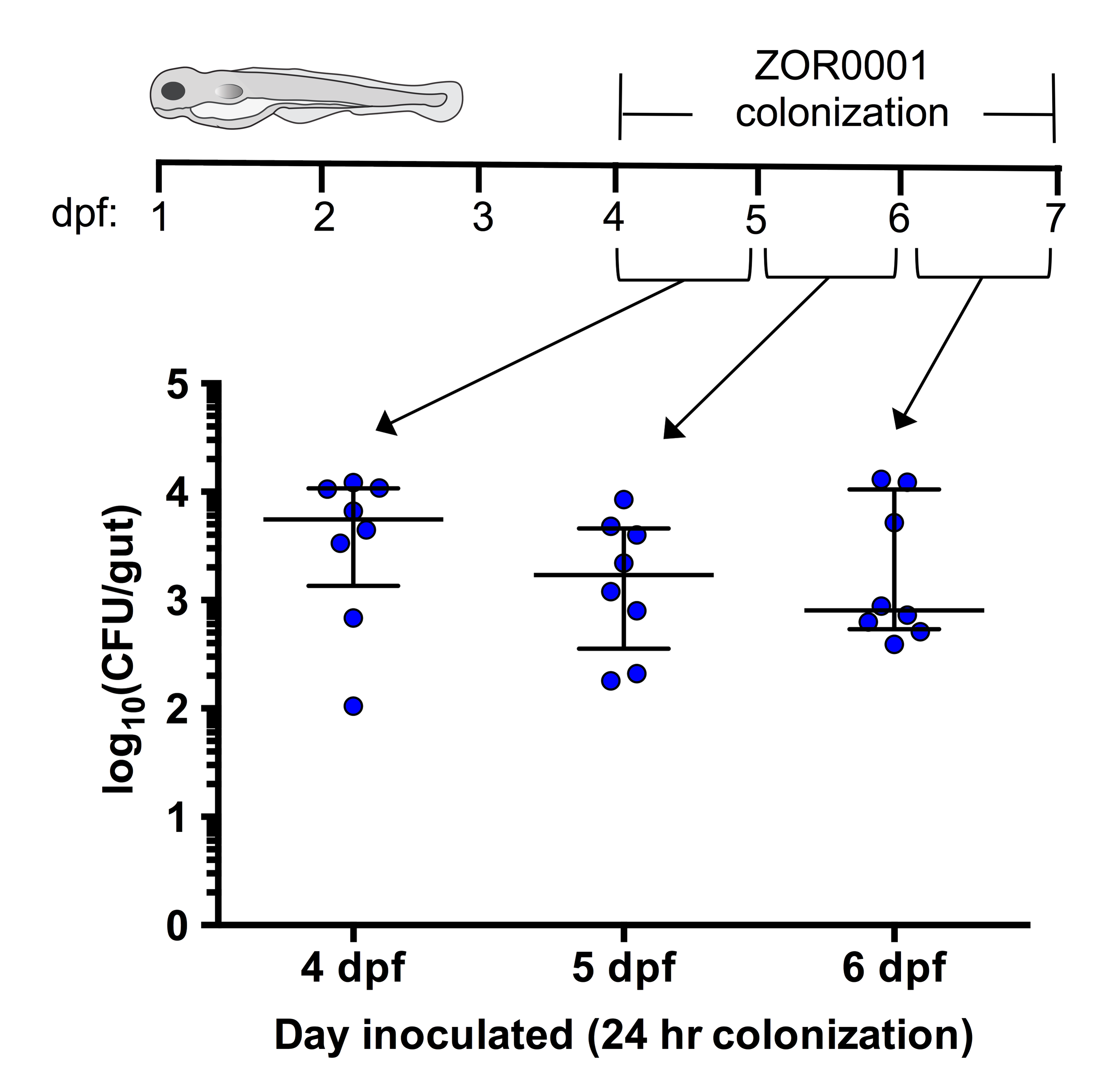

Supplement: S4 Fig — GF larval zebrafish were inoculated at 4, 5, or 6 dpf and then dissected at 24 hours post inoculation, and the intestines were plated to determine CFU/gut. Bars represent the median and interquartile ranges. Underlying data are provided in S1 Data. CFU/gut, colony-forming unit per gut; dpf, days post fertilization; GF, germ-free. (TIF) [file pbio.2006893.s007.tif]

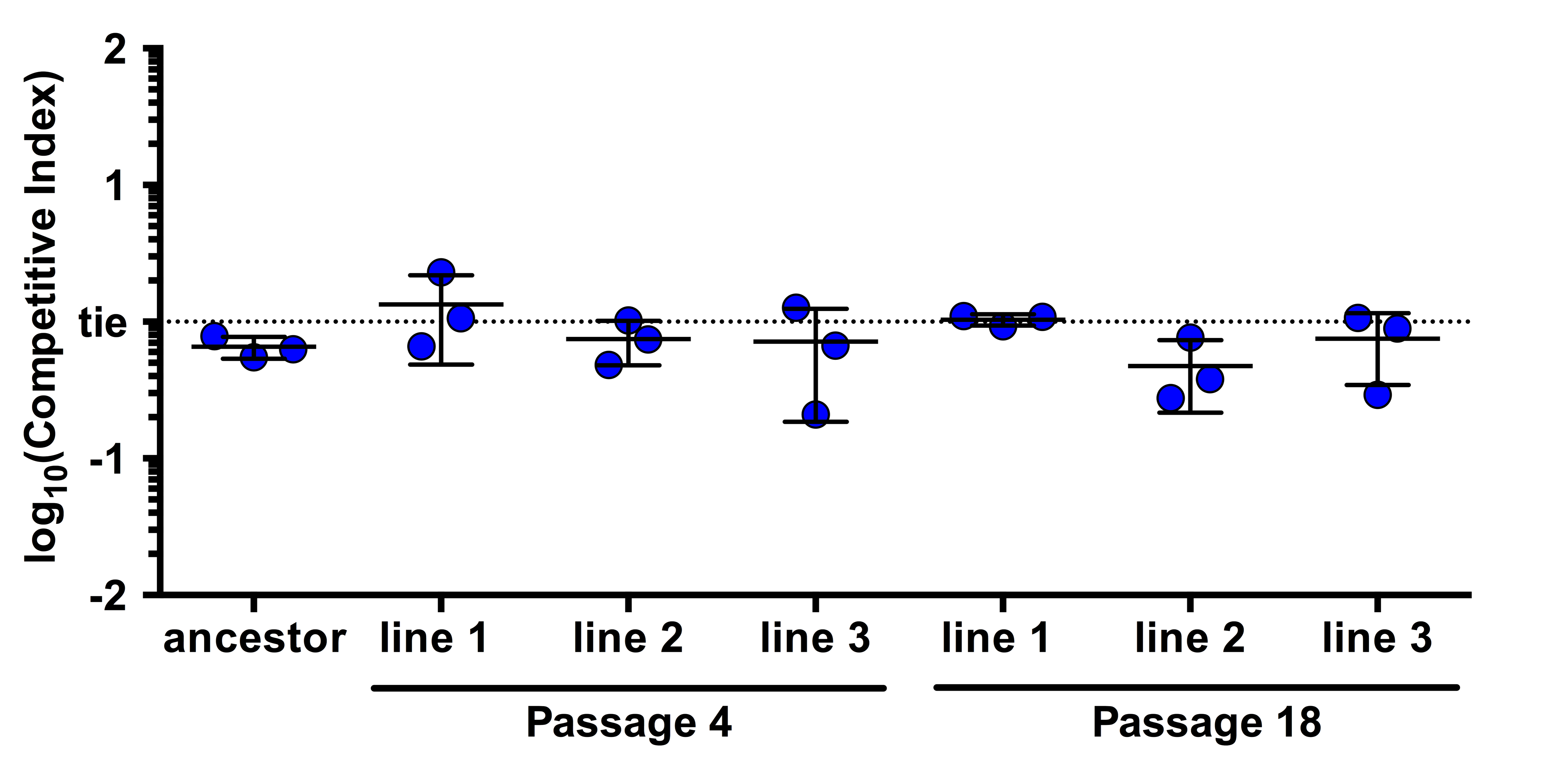

Supplement: S5 Fig — CIs of evolved isolates and the ancestor when competed against a differentially tagged ancestral strain in vitro. Each circle represents a CI from an independent biological replicate. Underlying data are provided in S1 Data. CI, competitive index. (TIF) [file pbio.2006893.s008.tif]

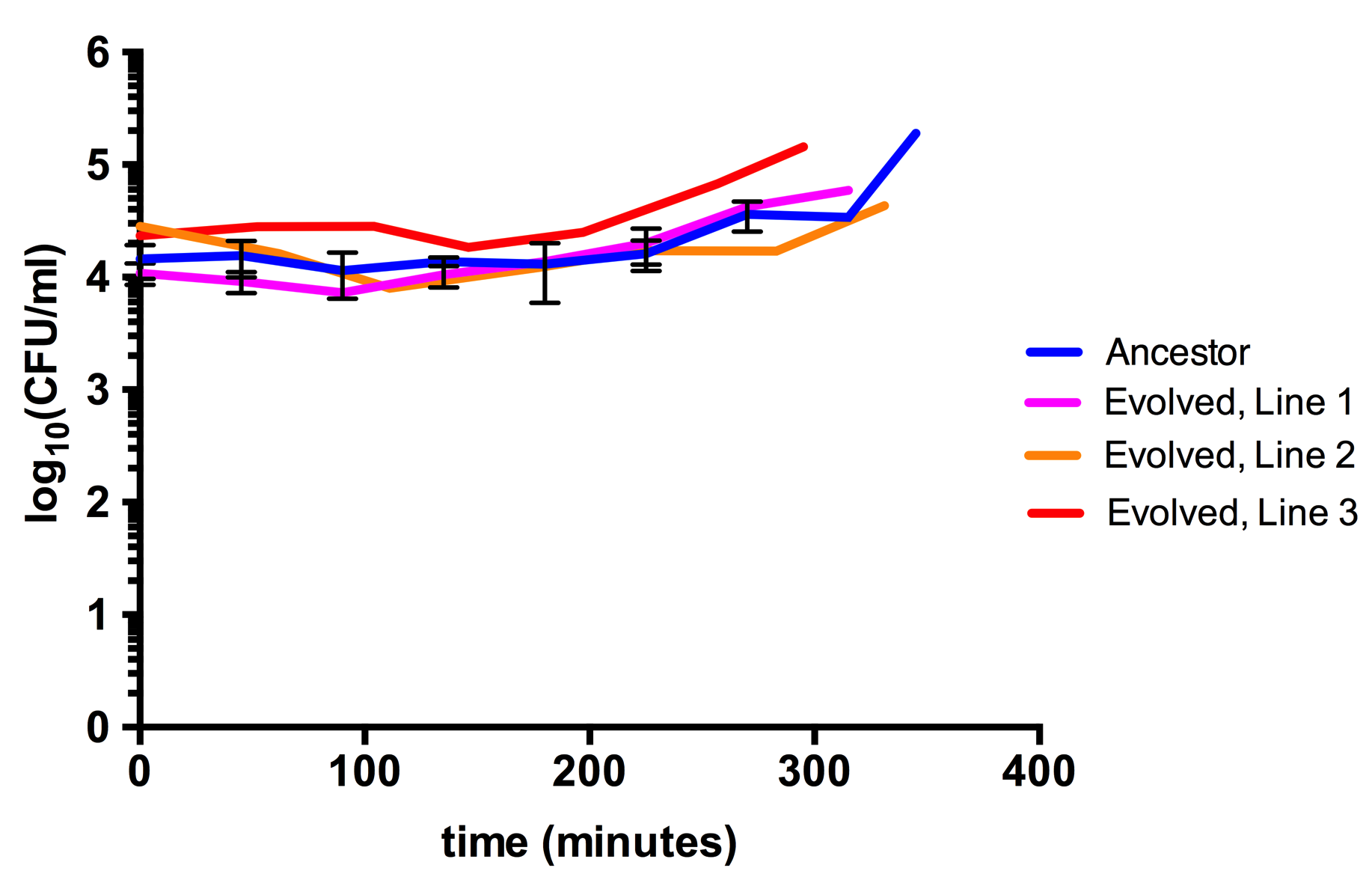

Supplement: S6 Fig — Data combined from three (ancestor), two (evolved, line 1), or one (evolved, lines 2 and 3) independent experiments; means (± SEM) are plotted for ancestor and line 1. Underlying data are provided in S1 Data. EM, embryo medium. (TIFF) [file pbio.2006893.s009.tiff]

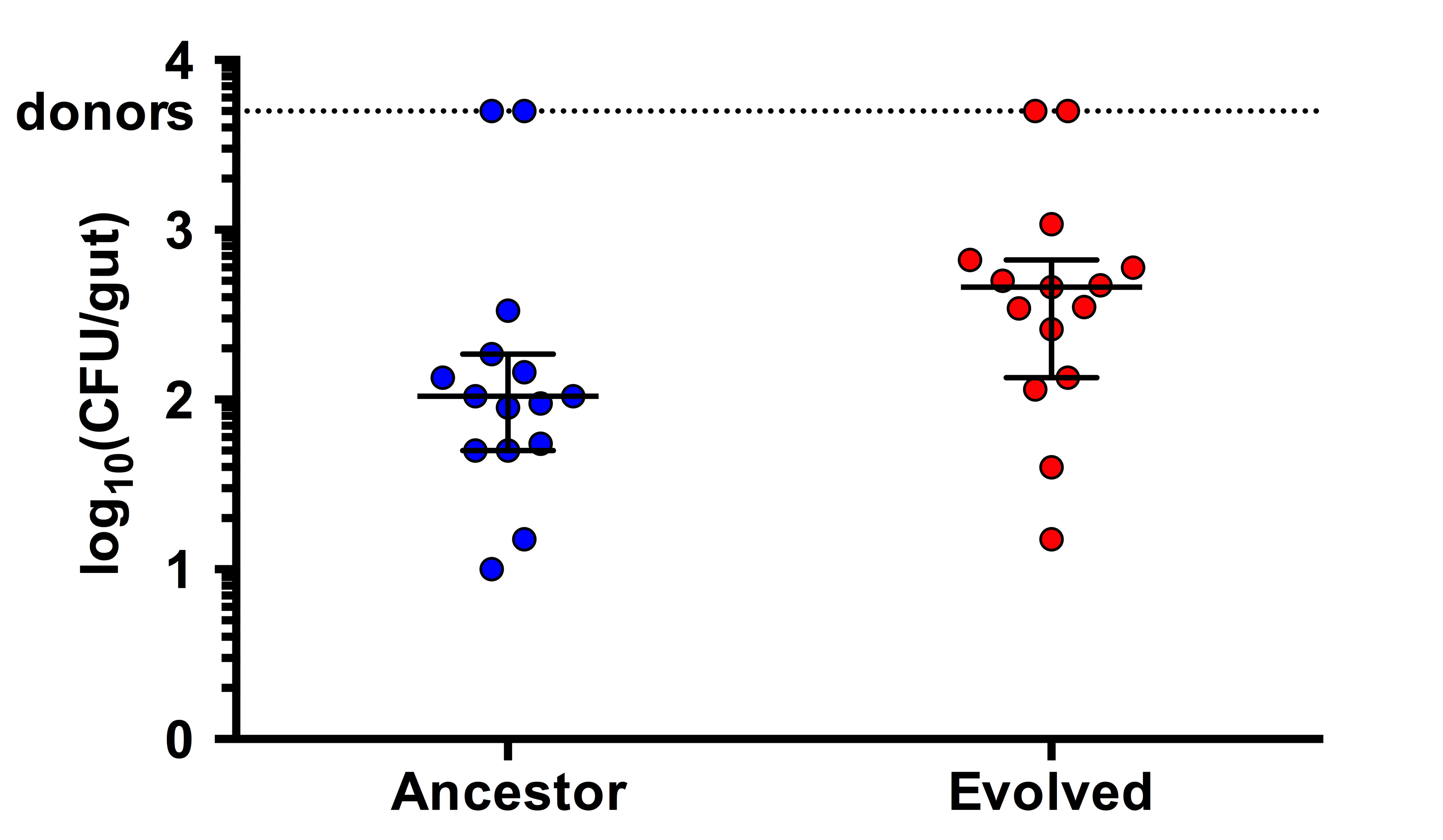

Supplement: S7 Fig — Mono-associated “donor” fish were incubated with 13 GF “recipient” fish for 12 hours, the guts were dissected, and the abundance of Aer01 in the fish was determined. p-Value = 0.003, determined using two-tailed Student’s t test, excluding the donor fish points. Underlying data are provided in S1 Data. GF, germ-free. (TIF) [file pbio.2006893.s010.tif]

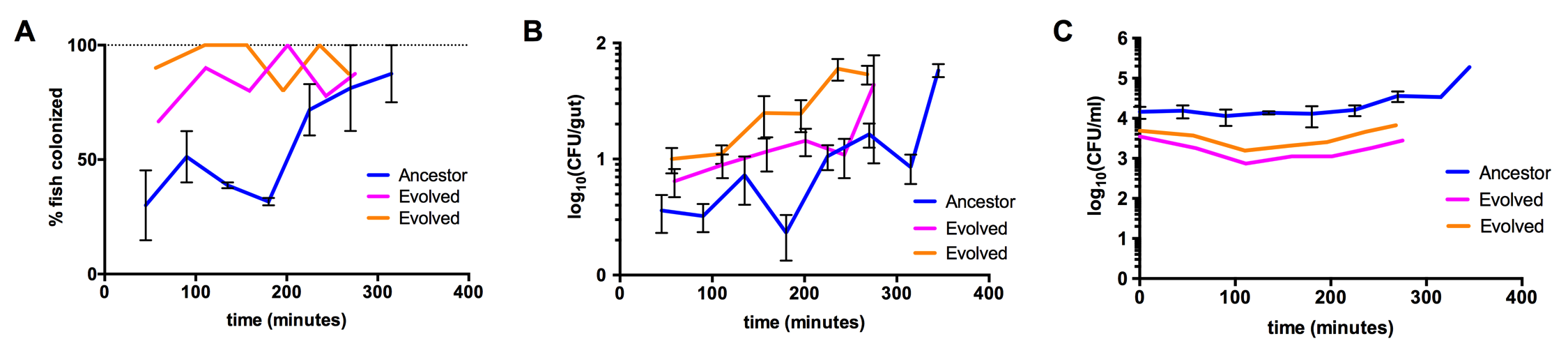

Supplement: S8 Fig — (A) Groups of mono-associated fish were dissected and plated about every 45 minutes, and the fraction of colonized hosts was determined. A higher proportion of fish are colonized at earlier time points for the evolved isolates compared to the ancestor. Mean (± SEM) is plotted for ancestor; ancestor data are the same as those plotted in Fig 4A. (B) CFU/gut (mean ± SEM) from the same samples presented in A show higher gut abundance for the evolved isolates. Ancestor data are the same as those plotted in Fig 4B. (C) EM abundance (CFU/ml) shows strains do not have differences in survival in flask EM. Mean (± SEM) is plotted for ancestor. Data combined from three (ancestor) or one (evolved) independent experiment. Underlying data for A–C are provided in S1 Data. CFU/gut, colony-forming unit per gut; EM, embryo medium. (TIFF) [file pbio.2006893.s011.tiff]

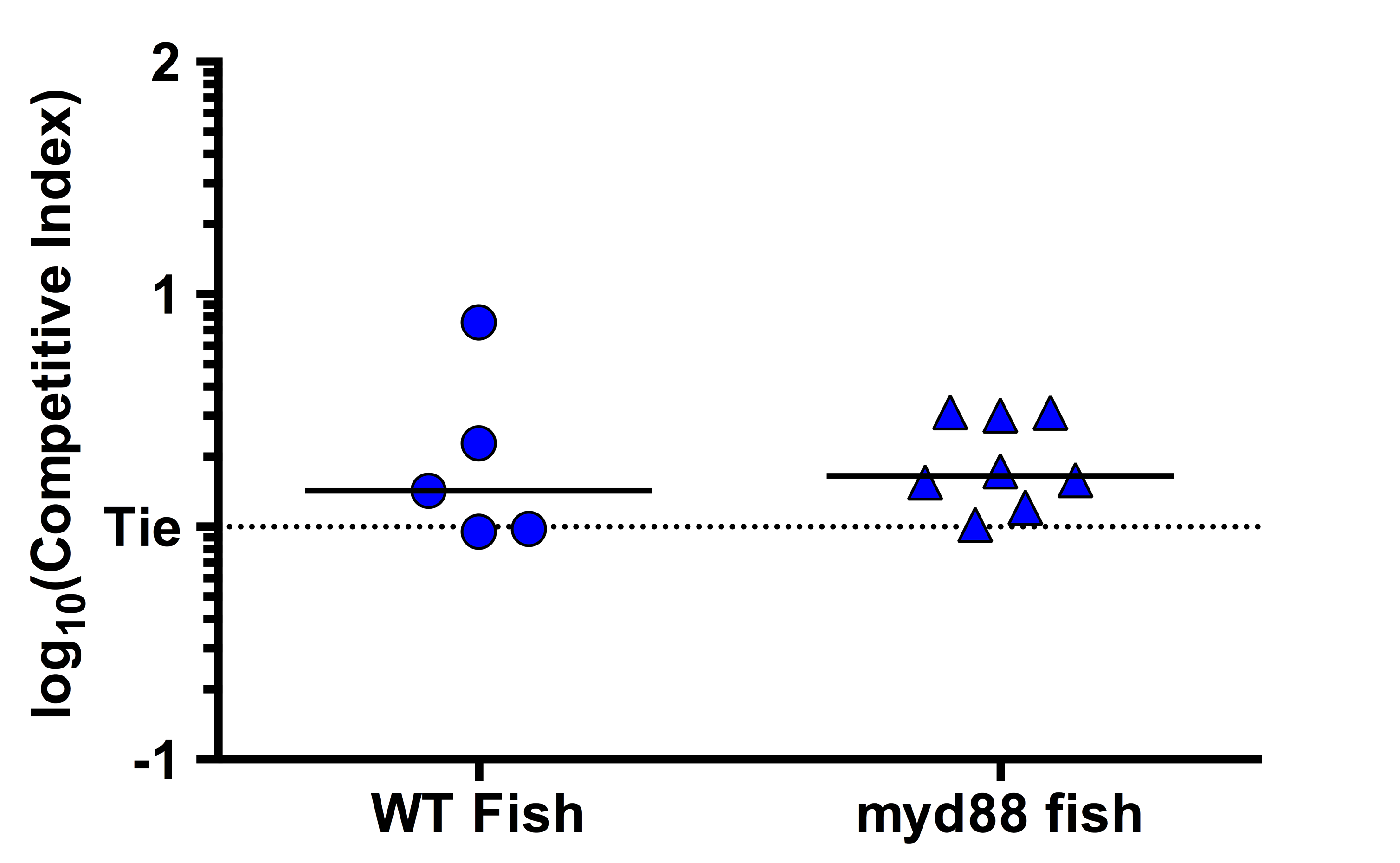

Supplement: S9 Fig — CI = (ancestor,evolved/ref)end/(ancestor,evolved/ref)start. Each data point represents a CI from an individual fish. Line = median. Underlying data are provided in S1 Data. CI, competitive index; WT, wild-type. (TIF) [file pbio.2006893.s012.tif]

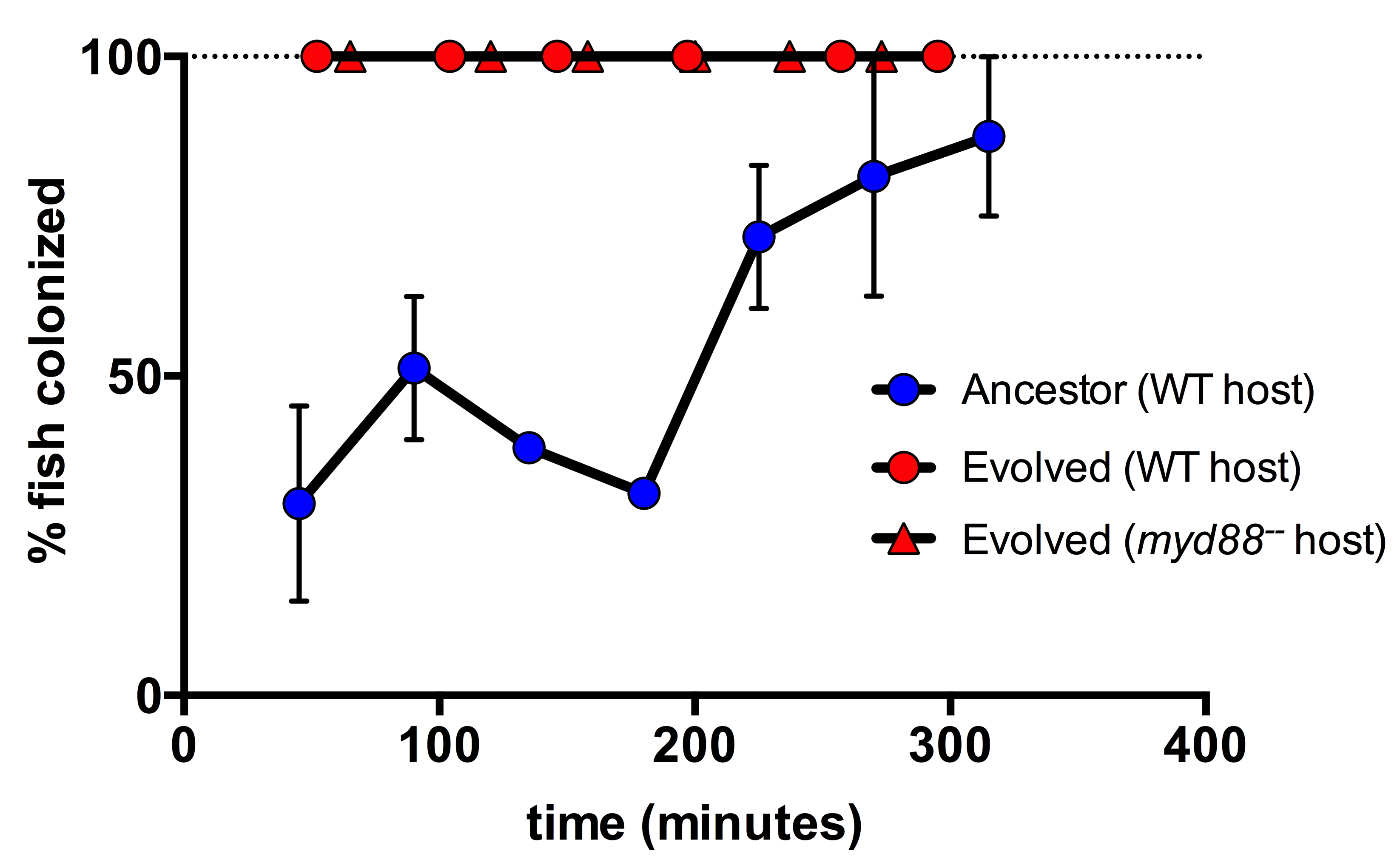

Supplement: S10 Fig — A higher proportion of fish are colonized at earlier time points for the evolved isolate compared to the ancestor. Mean (± SEM) is plotted for ancestor. Data combined from three (ancestor) or one (evolved) independent experiment. Ancestor data are the same as those plotted in Fig 4A. Underlying data are provided in S1 Data. (TIF) [file pbio.2006893.s013.tif]

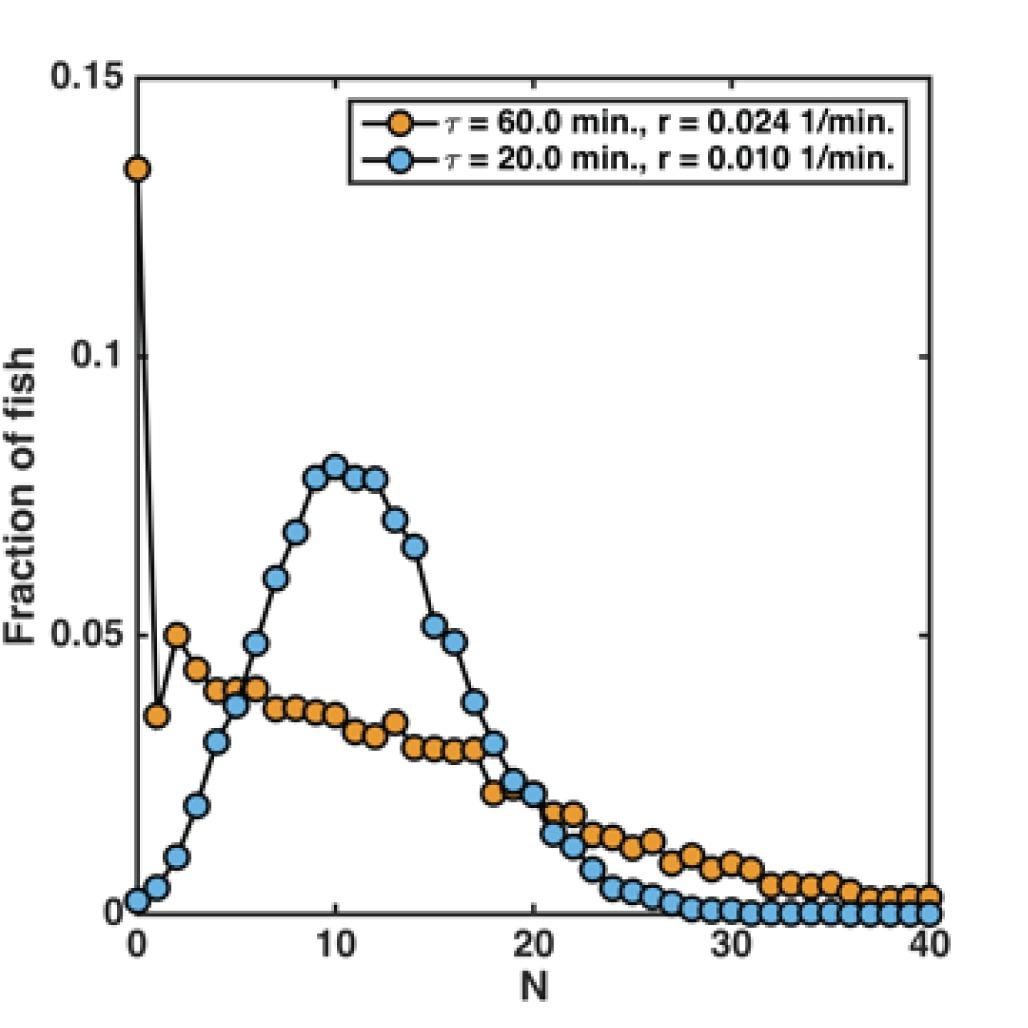

Supplement: S11 Fig — Histograms of bacterial population (N) across 10,000 fish at t = 120 minutes, with two different parameter values characterizing the migration and growth rates, each of which give the same mean population (N = 12) but show very different distributions. Note the large fraction of uncolonized fish (N = 0) if the mean entry time (τ) is large. Underlying data are provided in S1 Data. (TIF) [file pbio.2006893.s014.tif]

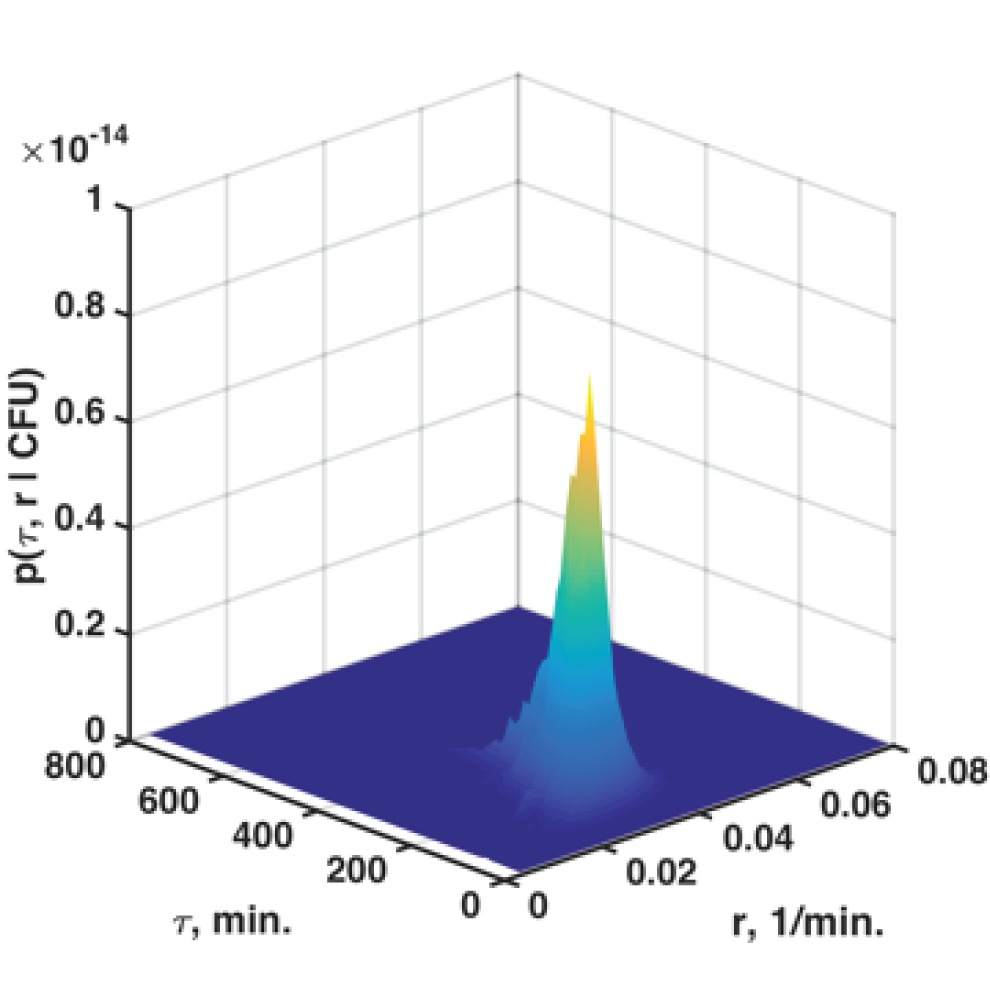

Supplement: S12 Fig — Underlying data are provided in S1 Data. CFU, colony-forming unit. (TIF) [file pbio.2006893.s015.tif]
